# Application of randomized quadrature formulas to the finite element method for elliptic equations

**DOI:** 10.1007/s10543-026-01126-8

**Published:** 2026-04-22

**Authors:** Raphael Kruse, Nick Polydorides, Yue Wu

**Affiliations:** 1 Institut für Mathematik, Martin-Luther-Universität Halle-Wittenberg, Halle (Saale), 06099 Germany; 2 School of Engineering, University of Edinburgh, Edinburgh, EH9 3FB UK; 3 Department of Mathematics and Statistics, University of Strathclyde, Glasgow, G1 1XH UK

**Keywords:** Finite element method, Monte Carlo methods, Quadrature formulas, Elliptic partial differential equations, 65C05, 65D32, 65N30

## Abstract

The implementation of the finite element method for linear elliptic equations requires to assemble the stiffness matrix and the load vector. In general, the entries of this matrix-vector system are not known explicitly but need to be approximated by quadrature rules. If the coefficient functions of the differential operator or the forcing term are irregular, then standard quadrature formulas, such as the barycentric quadrature rule, may not be reliable. In this paper we investigate the application of two randomized quadrature formulas to the finite element method for such elliptic boundary value problems with irregular coefficient functions. We give a detailed error analysis of these methods, discuss their implementation, and demonstrate their capabilities in several numerical experiments.

## Introduction

Let ${\mathcal {D}}\subset {\mathbb {R}}^2$ be a convex, bounded, and polygonal domain. We consider a linear elliptic boundary value problem of the following form: Find a mapping $u :{\mathcal {D}}\rightarrow {\mathbb {R}}$ such that

1.1 $$\begin{aligned} {\left\{ \begin{array}{ll} -\textrm{div}\big ( \sigma \nabla u \big ) = f,&  \text { in } {\mathcal {D}},\\ u = 0, &  \text { on } \partial {\mathcal {D}}, \end{array}\right. } \end{aligned}$$

where $\sigma , f :{\mathcal {D}}\rightarrow {\mathbb {R}}$ are given coefficient functions with $\sigma (x) \ge \sigma _0 > 0$ for all $x \in {\mathcal {D}}$. Provided $\sigma$ is globally bounded and *f* is square-integrable, it is well-known that (1.1) admits a unique solution $u \in H^1_0({\mathcal {D}})$ in the weak sense satisfying

1.2 $$\begin{aligned} \int _{\mathcal {D}}\sigma (x) \nabla u(x) \cdot \nabla v(x) \,\textrm{d}x = \int _{\mathcal {D}}f(x) v(x) \,\textrm{d}x \end{aligned}$$

for all $v \in H^1_0({\mathcal {D}})$. Here, we denote by $H^1_0({\mathcal {D}})$ the Sobolev space of weakly differentiable and square-integrable functions which (in some sense) satisfy the homogeneous Dirichlet boundary condition. In Section 2 we provide more details on the function spaces used throughout this paper. We also refer, for instance, to [5, Chapters 8–9] or [11, Chapter 6] for an introduction to the variational formulation of elliptic boundary value problems of the form (1.1).

Elliptic equations such as (1.1) appear in many applications, e.g., in mechanical engineering and physics. It is also an intensively studied problem to introduce the Galerkin finite element method as found in many text books in numerical analysis, e.g. [4, 27, 28, 36]. In the same spirit, we use (1.1) as a model problem to demonstrate the applicability of randomized quadrature formulas to the finite element method.

To this end, we consider a family $(\mathcal {T}_h)_{h \in (0,1]}$ of finite subdivisions of the polygonal domain ${\mathcal {D}}\subset {\mathbb {R}}^2$ into triangles. Hereby, the parameter $h \in (0,1]$ denotes the maximal edge length of the elements in $\mathcal {T}_h$. For every partition $\mathcal {T}_h$ we define $S_h \subset H^1_0({\mathcal {D}})$ as the associated finite element space consisting of piecewise linear functions.

Then, we obtain an approximation of the exact solution to the boundary value problem (1.1) by solving the following finite dimensional problem: For $h \in (0,1]$ find $u_h \in S_h$ satisfying

1.3 $$\begin{aligned} \int _{\mathcal {D}}\sigma (x) \nabla u_h(x) \cdot \nabla v_h(x) \,\textrm{d}x = \int _{\mathcal {D}}f(x) v_h(x) \,\textrm{d}x \end{aligned}$$

for all $v_h \in S_h$. For the practical computation of the approximation $u_h \in S_h$, it is then convenient to rewrite (1.3) as a system of linear equations. More precisely, let $(\varphi _j)_{j = 1}^{N_h}$ be a basis of $S_h$, where $N_h = \dim (S_h)$ denotes the number of degrees of freedom. Then, we have the representation

$$\begin{aligned} u_h = \sum _{j = 1}^{N_h} u_j \varphi _j, \end{aligned}$$

where the entries of the vector $\textbf{u} = [u_1,\ldots ,u_{N_h}]^{\top } \in {\mathbb {R}}^{N_h}$ are yet to be determined. After inserting this representation of $u_h$ into the finite dimensional problem (1.3) and by testing with all basis functions $(\varphi _j)_{j = 1}^{N_h}$ we arrive at a system of linear equations. In matrix-vector form this system is written as

1.4 $$\begin{aligned} A_h \textbf{u} = f_h, \end{aligned}$$

where the *stiffness matrix* $A_h \in {\mathbb {R}}^{N_h \times N_h}$ is given by

1.5 $$\begin{aligned} [A_h]_{i,j} = \int _{\mathcal {D}}\sigma (x) \nabla \varphi _i(x) \cdot \nabla \varphi _j (x) \,\textrm{d}x \end{aligned}$$

for all $i,j \in \{1,\ldots ,N_h\}$. Moreover, the *load vector* $f_h \in {\mathbb {R}}^{N_h}$ has the entries

1.6 $$\begin{aligned} [f_h]_i = \int _{\mathcal {D}}f(x) \varphi _i(x) \,\textrm{d}x, \quad i \in \{1,\ldots , N_h\}. \end{aligned}$$

If, on the one hand, the entries of $A_h$ and $f_h$ are known explicitly, it is straight-forward to use standard solvers for the linear system (1.4) in order to determine $\textbf{u} \in {\mathbb {R}}^{N_h}$ and, hence, $u_h \in S_h$ numerically. For instance, we refer to the monograph [17] for an overview of suitable solvers.

On the other hand, for general $\sigma \in L^\infty ({\mathcal {D}})$ and $f \in L^2({\mathcal {D}})$, the entries of the stiffness matrix and the load vector are often not computable explicitly. Such irregular coefficients often appear in problems in uncertainty quantification to model incomplete knowledge of the problem parameters. See [2] and the references therein. In the literature, the reader is advised to approximate the entries by suitable quadrature formulas. For instance, we refer to [28, Section 5.6] and [36, Section 4.3].

However, standard methods for numerical integration, such as the trapezoidal sum, require point evaluations of the coefficient functions $\sigma$ and *f*. Therefore, these quadrature formulas are, in general, only applicable if additional smoothness requirements, such as continuity, are imposed on $\sigma$ and *f*. The purpose of this paper is to show that this problem can be circumvented if we approximate the entries of $A_h$ and $f_h$ by *randomized quadrature formulas*. As it will turn out, these quadrature formulas do not require the continuity of *f* and $\sigma$.

Before we give a more detailed outline of the content of this paper, let us mention that we consider randomized quadrature formulas of a form that has originally been introduced by S. Haber in [14–16]. His important observation was that the accuracy of the standard Monte Carlo method can be increased drastically, if the random sampling points are distributed more evenly over the integration domain. More precisely, he proposed to place the random sampling points in disjoint subdomains whose volumes decay asymptotically with the number of samples. If the integrand possesses more regularity than being merely square-integrable this approach reduces the variance of the randomized quadrature formula significantly. In particular, one often observes an higher order of convergence compared to standard Monte Carlo estimators or purely deterministic methods. For more details on this line of arguments we also refer to the proof of Lemma 1 further below. Moreover, related results are found in [6, 30].

More recently, it has been shown that such randomized quadrature formulas are also applicable to the numerical approximation of ordinary differential equations with time-irregular coefficient functions. We refer, for instance, to [8, 19, 21, 25, 34, 35] for results on randomized one-step methods. Further, these methods have also been applied for the *temporal discretization* of evolution equations in infinite dimensions, see [10, 20], and of stochastic differential equations, see [26, 32].

Besides [18], where the information based complexity of randomized algorithms for elliptic partial differential equations has been investigated, it appears that the application of randomized quadrature formulas to the *spatial discretization* of boundary value problems is not well-studied yet.

In this paper, we first consider a stratified Monte Carlo estimator in the spirit of [14]. More precisely, the estimator defined in (3.19) below, is based on an admissible triangulation $\mathcal {T}_h$ of ${\mathcal {D}}$ and exactly one uniformly distributed random point on each triangle of the triangulation. We show in Section 3 that this estimator gives approximations of the entries in the stiffness matrix and the load vector, which are convergent at least with order 1 with respect to the root-mean-square norm. Under slightly increased regularity assumptions, such as $f \in L^p({\mathcal {D}})$ with $p \in (2,\infty ]$ and $\sigma \in W^{s,q}({\mathcal {D}})$ with $s \in (0,1]$, $q \in (2,\infty ]$, we also show that the resulting randomized finite element solution $u_h^{\text {MC}}$ converges to the exact solution $u \in H^1_0({\mathcal {D}})$. The precise error estimate is given in Theorem 2.

In Section 4, we propose an importance sampling estimator for the approximation of the load vector. Hereby, the random points are placed according to a non-uniform distribution, whose probability density function is proportional to the basis functions of the finite element space. The section also contains a detailed analysis of the error with respect to the norms in $L^2({\mathcal {D}})$ and $H^1_0({\mathcal {D}})$, where we purely focus on the associated finite element problem for the Poisson equation (4.41), i.e. Equation (1.1) with $\sigma \equiv 1$. These results are stated in Theorem 4 and Theorem 5.

In Section 5 we discuss the implementation of the randomized quadrature formulas. Essentially, this is achieved by a transformation to a reference triangle, typically the 2-simplex, and a general rejection algorithm. Finally, we report on some numerical experiments in Section 6.

## Notation and preliminaries

In this section, we fix some notation and introduce several function spaces, which are used throughout this paper. We also revisit the variational formulation of the boundary value problem (1.1) and its approximation by the finite element method. The section also contains a brief overview of some terminology from probability.

By ${\mathbb {N}}$ we denote the set of all positive integers, while ${\mathbb {N}}_0 := {\mathbb {N}}\cup \{0\}$. As usual, the set ${\mathbb {R}}$ consists of all real numbers. By $| \cdot |$ we denote the Euclidean norm on the Euclidean space ${\mathbb {R}}^d$ for any $d \in {\mathbb {N}}$. In particular, if $d = 1$ then $| \cdot |$ coincides with taking the absolute value.

Throughout this paper we often use *C* as a generic constant, which may vary from appearance to appearance. However, *C* is not allowed to depend on numerical parameters such as $h \in (0,1]$.

Next, let us introduce some function spaces. Throughout this paper, we assume that ${\mathcal {D}}\subset {\mathbb {R}}^2$ is a bounded, convex and polygonal domain. By $L^p({\mathcal {D}})$, $p \in [1,\infty ]$, we denote the Banach space of (equivalence classes of) *p*-fold Lebesgue integrable functions, which is endowed with the norm

$$\begin{aligned} \Vert { g} \Vert _{L^p({\mathcal {D}})}&= \Big ( \int _{\mathcal {D}}|{ g}(x)|^p \,\textrm{d}x \Big )^{\frac{1}{p}} \quad \text { for } p \in [1,\infty ),\\ \Vert { g} \Vert _{L^\infty ({\mathcal {D}})}&= \mathop {\mathrm {ess\,sup}}\limits _{x \in {\mathcal {D}}} |{ g}(x)|. \end{aligned}$$

As it is customary, we do not distinguish notationally between functions and their equivalence classes.

An important example of an element in $L^p({\mathcal {D}})$ for any value of $p \in [1,\infty ]$ is the indicator function of a measurable set $B \subseteq {\mathcal {D}}$ denoted by $\mathbb {I}_B$. This function fulfills $\mathbb {I}_B(x) = 1$ if $x \in B$, else $\mathbb {I}_B(x)=0$.

Moreover, we denote by $W^{k,p}({\mathcal {D}}) \subset L^p({\mathcal {D}})$, $p \in [1,\infty ]$, $k \in {\mathbb {N}}$, the Sobolev space with differentiation index *k*. To be more precise, $W^{k,p}({\mathcal {D}})$ consists of all *p*-fold integrable functions that are *k*-times partially differentiable in the weak sense and whose derivatives are also *p*-fold integrable. If $W^{k,p}({\mathcal {D}})$ is endowed with the norm

$$\begin{aligned} \Vert { g} \Vert _{W^{k,p}({\mathcal {D}})}&= \Big ( \sum _{ \alpha \in {\mathbb {N}}_0^2, |\alpha |\le k} \Vert \partial ^\alpha { g} \Vert _{L^p({\mathcal {D}})}^p \Big )^{\frac{1}{p}} \quad \text { for } p \in [1,\infty ),\\ \Vert { g} \Vert _{W^{k,\infty }({\mathcal {D}})}&= \sum _{ \alpha \in {\mathbb {N}}_0^2, |\alpha |\le k} \Vert \partial ^\alpha { g} \Vert _{L^\infty ({\mathcal {D}})}, \end{aligned}$$

then it is also a Banach space. Here we make use of the standard multi-index notation for partial derivatives, that is, for $\alpha \in {\mathbb {N}}_0^2$ we define $|\alpha | = \alpha _1 + \alpha _2$ and

$$\begin{aligned} \partial ^\alpha { g} := \frac{\partial ^{|\alpha |}}{\partial _{x_1}^{\alpha _1} \partial _{x_2}^{\alpha _2}} { g}. \end{aligned}$$

Further, if $p = 2$ then $L^2({\mathcal {D}})$ and $H^k({\mathcal {D}}):= W^{k,2}({\mathcal {D}})$ are Hilbert spaces. The inner products are denoted by $(\cdot , \cdot )_{L^2({\mathcal {D}})}$ and $(\cdot , \cdot )_{H^k({\mathcal {D}})}$, respectively.

In order to incorporate homogeneous Dirichlet boundary conditions, we also introduce the space $H^1_0({\mathcal {D}})$, which is defined as the closure of the set of all infinitely often differentiable functions with compact support in ${\mathcal {D}}$ with respect to the norm in $H^1({\mathcal {D}})$, that is

$$\begin{aligned} H^1_0({\mathcal {D}}) := \overline{ C_{c}^\infty ({\mathcal {D}})}^{\Vert \cdot \Vert _{H^1({\mathcal {D}})}}. \end{aligned}$$

It is well-known that the standard $H^1({\mathcal {D}})$-norm and the semi-norm

$$\begin{aligned} | { g} |_{H^1({\mathcal {D}})} = \Big ( \sum _{ i = 1}^2 \Big \Vert \frac{\partial }{\partial x_i} { g} \Big \Vert _{L^2({\mathcal {D}})}^2 \Big )^{\frac{1}{2}} = \Big ( \int _{\mathcal {D}}| \nabla { g} |^2 \,\textrm{d}x \Big )^{\frac{1}{2}} \end{aligned}$$

are equivalent on $H^1_0({\mathcal {D}})$. In particular, the space $(H^1_0({\mathcal {D}}),|\cdot |_{H^1({\mathcal {D}})}, (\cdot ,\cdot )_{H^1_0({\mathcal {D}})})$ is a separable Hilbert space. For a detailed introduction to Sobolev spaces we refer the reader, for instance, to [11, Chapter 5].

For a domain ${\mathcal {D}}\subset {\mathbb {R}}^2$, $p \in [1,\infty )$, and $s \in (0,1)$ the Sobolev–Slobodeckij norm $\Vert \cdot \Vert _{W^{s,p}({\mathcal {D}})}$ is given by

2.7 $$\begin{aligned} \Vert { g} \Vert _{W^{s,p}({\mathcal {D}})} = \Big ( \Vert { g} \Vert ^p_{L^p({\mathcal {D}})} + \int _{{\mathcal {D}}} \int _{{\mathcal {D}}} \frac{|{ g}(x_1) - { g}(x_2) |^p}{|x_1 - x_2|^{2 + s p}} \,\textrm{d}x_2 \,\textrm{d}x_1 \Big )^{\frac{1}{p}}. \end{aligned}$$

Then, the fractional order Sobolev space $W^{s,p}({\mathcal {D}})$ consists of all ${ g} \in L^p({\mathcal {D}})$ satisfying $\Vert { g} \Vert _{W^{s,p}({\mathcal {D}})}< \infty$. By $|\cdot |_{W^{s,p}({\mathcal {D}})}$ we denote the corresponding semi-norm, which only consists of the double integral part in (2.7). Further details on these spaces are found in [9].

Next, we revisit the variational formulation of the boundary value problem (1.1). If $\sigma \in L^\infty ({\mathcal {D}})$, $\sigma (x) \ge \sigma _0 > 0$ for almost every $x \in {\mathcal {D}}$, and $f \in L^2({\mathcal {D}})$, then it is well-known that the bilinear form $a :H^1_0({\mathcal {D}}) \times H^1_0({\mathcal {D}}) \rightarrow {\mathbb {R}}$ and the linear functional $F :H^1_0({\mathcal {D}}) \rightarrow {\mathbb {R}}$ given by

2.8 $$\begin{aligned} a(u,v)&:= \int _{\mathcal {D}}\sigma (x) \nabla u(x) \cdot \nabla v(x) \,\textrm{d}x,\end{aligned}$$

2.9 $$\begin{aligned} F(v)&:= \int _{\mathcal {D}}f(x) v(x) \,\textrm{d}x \end{aligned}$$

for all $u, v \in H^1_0({\mathcal {D}})$ are well-defined. Moreover, *a* is strongly positive and bounded, that is, it holds

2.10 $$\begin{aligned} a(v,v)&\ge \sigma _0 |v|_{H^1({\mathcal {D}})}^2,\end{aligned}$$

2.11 $$\begin{aligned} |a (u,v) |&\le \Vert \sigma \Vert _{L^\infty ({\mathcal {D}})} |u|_{H^1({\mathcal {D}})} |v|_{H^1({\mathcal {D}})} \end{aligned}$$

for all $u, v \in H^1_0({\mathcal {D}})$. Further, *F* is a bounded linear functional.

Therefore, the lemma of Lax–Milgram, cf. [11, Chapter 6], is applicable and ensures the existence of a unique weak solution $u \in H^1_0({\mathcal {D}})$ satisfying

2.12 $$\begin{aligned} a(u,v) = F(v) \quad \text {for all } v \in H^1_0({\mathcal {D}}). \end{aligned}$$

Observe that (2.12) coincides with (1.2).

For the error analysis in Section 3 and Section 4, it will be necessary to impose the following additional regularity condition on the exact solution.

### Assumption 1

The variational problem (2.12) admits a uniquely determined strong solution, i.e., the unique weak solution *u* to (2.12) is an element of $H^1_0({\mathcal {D}}) \cap H^2({\mathcal {D}})$.

We refer, for instance, to [13, Theorem 3.2.1.2], which gives sufficient conditions for the existence of a strong solution. For example, if ${\mathcal {D}}$ is a convex, bounded and open subset of ${\mathbb {R}}^2$ and if $\sigma \in L^\infty ({\mathcal {D}})$ has a globally Lipschitz continuous extension on $\overline{{\mathcal {D}}}$, then Assumption 1 is satisfied for every $f \in L^2({\mathcal {D}})$.

Next, we briefly review the finite element method for problem (1.1). To this end, let $(\mathcal {T}_h)_{h \in (0,1]}$ be a family of admissible triangulations of ${\mathcal {D}}$. More precisely, for every $h \in (0,1]$ it holds that each triangle $T \in \mathcal {T}_h$ is an open subset of ${\mathcal {D}}$ satisfying

$$\begin{aligned} \bigcup _{T \in \mathcal {T}_{h}} \overline{T}&= \overline{{\mathcal {D}}} \quad \text { and } T \cap T' = \emptyset , \quad \text {for all } T,T' \in \mathcal {T}_h, T \ne T'. \end{aligned}$$

Further, it is assumed that no vertex of any triangle lies in the interior of an edge of any other triangle of the triangulation, cf. [4, Definition 3.3.11]. Typically, the parameter $h \in (0,1]$ denotes the maximal edge length of all triangles in $\mathcal {T}_h$. Moreover, the area of a triangle *T* is denoted by |*T*|.

As usual, we define the finite element space $S_h$ associated to a triangulation $\mathcal {T}_h$, $h \in (0,1]$, by

$$\begin{aligned} S_h = \{ v_h \in C(\overline{{\mathcal {D}}})\, : \, v_h = 0 \, \text { on } \partial {\mathcal {D}}, \, v_h|_{T} \in \varPi _1 \, \forall T \in \mathcal {T}_h\}. \end{aligned}$$

Hereby, the set $\varPi _1$ consists of all polynomials up to degree 1. The finite element space $S_h$ is finite dimensional and $N_h = \dim (S_h)$ is called the *number of degrees of freedom*. It coincides with the number of interior nodes $(z_i)_{i = 1}^{N_h}$ of the triangulation. By $(\varphi _j)_{j = 1}^{N_h} \subset S_h$ we denote the standard Lagrange basis of $S_h$ determined by $\varphi _j(z_i) = \delta _{i,j}$ for all $i,j = 1,\ldots ,N_h$. Further details on the construction of finite element spaces are found, e.g., in [4, Chapter 3] or [28, Chapter 5].

For the error analysis in Section 3 and Section 4 we have to impose the following additional condition on the family of triangulations.

### Assumption 2

We assume that $(\mathcal {T}_h)_{h \in (0,1]}$ is a family of admissible and quasi-uniform triangulations. In particular, the interior angles of each triangle in $\mathcal {T}_h$ are bounded from below by a positive constant, independently of *h*. In addition, there exists $c \in (0,\infty )$ such that for every $h \in (0,1]$ and $T \in \mathcal {T}_h$ it holds that $|T| \ge c h^2$.

The assumption enables us to make use of a maximum norm estimate for functions from the finite element space $S_h$, which we cite from [37, Lemma 6.4]: If Assumption 2 is satisfied then there exists $C \in (0,\infty )$, independently of $h \in (0,1]$, such that

2.13 $$\begin{aligned} \Vert v_h \Vert _{L^\infty ({\mathcal {D}})} \le C \ell _h^{\frac{1}{2}} | v_h |_{H^1({\mathcal {D}})} \end{aligned}$$

for every $v_h \in S_h$, where $\ell _h = \max (1, \log (1/h))$.

Further, we recall that for a quasi-uniform family of triangulations the following inverse estimate is satisfied

2.14 $$\begin{aligned} | v_h |_{H^1({\mathcal {D}})} \le C h^{-1} \Vert v_h \Vert _{L^2({\mathcal {D}})} \end{aligned}$$

for every $v_h \in S_h$, where *C* is independent of the triangulation $\mathcal {T}_h$. For a proof of (2.14) we refer to [4, Section 4.5].

Next, we introduce the *Ritz projector* $R_h :{H}^{1}_0({\mathcal {D}}) \rightarrow S_h$ as the orthogonal projector onto $S_h$ with respect to the bilinear form *a*. To be more precise, as a consequence of the lemma of Lax–Milgram, for each $v \in {H}^{1}_0({\mathcal {D}})$ there exists a unique element $R_h v \in S_h$ fulfilling

2.15 $$\begin{aligned} a(R_h v, v_h) = a(v,v_h) \quad \text { for all } v_h \in S_h. \end{aligned}$$

Note that $R_h :H^1_0({\mathcal {D}}) \rightarrow S_h$ is a bounded linear operator. In addition, there exists $C \in (0,\infty )$ such that for every $h \in (0,1]$ and $v \in H^1_0({\mathcal {D}}) \cap H^2({\mathcal {D}})$ it holds

2.16 $$\begin{aligned} | (R_h - I) v|_{H^1({\mathcal {D}})}&\le C h \Vert v\Vert _{H^2({\mathcal {D}})},\end{aligned}$$

2.17 $$\begin{aligned} \Vert (R_h - I)v \Vert _{L^2({\mathcal {D}})}&\le C h^2 \Vert v\Vert _{H^2({\mathcal {D}})}. \end{aligned}$$

A proof is found, for instance, in [28, Theorem 5.5].

For the introduction and the error analysis of Monte Carlo methods, we also require some fundamental concepts from probability and stochastic analysis. For a general introduction readers are referred to standard monographs on this topic, for instance [23, 24]. For the measure theoretical background see also [3, 7].

First, let us recall that a *probability space* $(\varOmega ,\mathcal {F},{\mathbb {P}})$ consists of a measurable space $(\varOmega ,\mathcal {F})$ endowed with a finite measure ${\mathbb {P}}$ satisfying ${\mathbb {P}}(\varOmega ) = 1$. The value ${\mathbb {P}}(A) \in [0,1]$ is interpreted as the *probability* of the *event* $A \in {\mathcal {F}}$. A mapping $X :\varOmega \rightarrow {\mathbb {R}}^d$, $d \in {\mathbb {N}}$, is called a *random variable* if *X* is ${\mathcal {F}}/ \mathcal {B}({\mathbb {R}}^d)$-measurable, where $\mathcal {B}({\mathbb {R}}^d)$ denotes the Borel-$\sigma$-algebra generated by the set of all open subsets of ${\mathbb {R}}^d$. More precisely, it holds true that

$$\begin{aligned} X^{-1}(B)= \big \{ \omega \in \varOmega \, : \, X(\omega )\in B \big \} \in \mathcal {F} \end{aligned}$$

for all $B \in \mathcal {B}({\mathbb {R}}^d)$. Every random variable induces a probability measure on its image space. In fact, the measure ${\mathbb {P}}_X :\mathcal {B}({\mathbb {R}}^d) \rightarrow [0,1]$ given by ${\mathbb {P}}_X(B)={\mathbb {P}}(X^{-1}(B))$ for all $B \in \mathcal {B}({\mathbb {R}}^d)$ is a probability measure on the measurable space $({\mathbb {R}}^d, \mathcal {B}({\mathbb {R}}^d))$. Usually, ${\mathbb {P}}_X$ is called the *distribution* of *X*.

If the distribution ${\mathbb {P}}_X$ is absolutely continuous with respect to the Lebesgue measure, then there exists a measurable, non-negative mapping $g_X :{\mathbb {R}}^d \rightarrow {\mathbb {R}}$ with

$$\begin{aligned} {\mathbb {P}}_X(B) = {\mathbb {P}}( X^{-1}(B) ) = \int _B g_X(x) \,\textrm{d}x \end{aligned}$$

for every $B \in \mathcal {B}({\mathbb {R}}^d)$. The mapping $g_X$ is called the *probability density function* of *X* and we write $X \sim g_X(x) \,\textrm{d}x$.

Next, let us recall that a random variable $X :\varOmega \rightarrow {\mathbb {R}}^d$ is called *integrable* if $\int _{\varOmega } |X(\omega )| \,\textrm{d}{\mathbb {P}}(\omega )<\infty$. Then, the *expectation* of *X* is defined as

$${\mathbb {E}}[X]:=\int _{\varOmega }X(\omega )\,\textrm{d}{\mathbb {P}}(\omega ) = \int _{{\mathbb {R}}^d} x \,\textrm{d}{\mathbb {P}}_{X}(x).$$

We say that *X* is centered if ${\mathbb {E}}[X] = 0$.

Moreover, we write $X \in L^p(\varOmega ;{\mathbb {R}}^d)$ with $p \in [1,\infty )$ if $\int _{\varOmega }|X(\omega )|^p \,\textrm{d}{\mathbb {P}}(\omega )<\infty$. If $d=1$, then we simply write $L^p(\varOmega ):= L^p(\varOmega ;{\mathbb {R}})$. In addition, the set $L^p(\varOmega ;{\mathbb {R}}^d)$ becomes a Banach space if we identify all random variables that only differ on a set of measure zero (i.e. probability zero) and if we endow $L^p(\varOmega ;{\mathbb {R}}^d)$ with the norm

$$\begin{aligned} \Vert X \Vert _{L^p(\varOmega ;{\mathbb {R}}^d)} = \big ( {\mathbb {E}}\big [ |X|^p \big ] \big )^{\frac{1}{p}} = \Big ( \int _{\varOmega } |X(\omega ) |^p \,\textrm{d}{\mathbb {P}}(\omega ) \Big )^{\frac{1}{p}}. \end{aligned}$$

In Section 3, we frequently encounter a family of $\mathcal {U}(T)$-distributed random variables $(Z_T)_{T \in \mathcal {T}_h}$. This means that for each $T \in \mathcal {T}$ the mapping $Z_T :\varOmega \rightarrow {\mathbb {R}}^2$ is a random variable that is *uniformly distributed* on the triangle *T*. More precisely, the distribution ${\mathbb {P}}_{Z_T}$ of $Z_T$ is given by ${\mathbb {P}}_{Z_T}(A) = \frac{|A \cap T|}{|T|}$ for every $A \in {\mathcal {B}}({\mathbb {R}}^2)$. Moreover, it follows from the transformation theorem that the expectation of $v \circ Z_T$ for an arbitrary function $v\in L^1({\mathcal {D}})$ is given by

$${\mathbb {E}}[v(Z_T)]= \int _{T} v(z)\frac{1}{|T|} \,\textrm{d}z = \int _{{\mathcal {D}}} v(z) \frac{1}{|T|} \mathbb {I}_T(z) \,\textrm{d}z,$$

where the mapping $g_{Z_T}(z) = \frac{1}{|T|} \mathbb {I}_{T}(z)$, $z \in {\mathcal {D}}\subset {\mathbb {R}}^2$, is the probability density function of $Z_T$.

Further, we say that a family of ${\mathbb {R}}^d$-valued random variables $(X_n)_{n \in {\mathbb {N}}}$ is *independent* if for any finite subset $M \subset {\mathbb {N}}$ and for arbitrary events $(A_m)_{m \in M} \subset \mathcal {B}({\mathbb {R}}^d)$ we have the multiplication rule

$$\begin{aligned} {\mathbb {P}}\Big ( \bigcap _{m \in M} \{ \omega \in \varOmega \, : \, X_m(\omega ) \in A_m \} \Big ) = \prod _{m \in M} {\mathbb {P}}\big ( \{ \omega \in \varOmega \, : \, X_m(\omega ) \in A_m \} \big ). \end{aligned}$$

On the level of distributions this basically means that the joint distribution of each finite subfamily $(X_m)_{m \in M}$ is equal to the product measure of the single distributions. This directly implies the multiplication rule for the expectation

2.18 $$\begin{aligned} {\mathbb {E}}\Big [ \prod _{m \in M} X_m \Big ] = \prod _{m \in M} {\mathbb {E}}\big [ X_m \big ], \end{aligned}$$

provided $X_m$ is integrable for each $m \in M$.

Finally, let us mention that we often encounter random variables taking values in a function space instead of ${\mathbb {R}}^d$. For instance, in Theorem 1 we construct a random variable with values in $S_h \subset H^1_0({\mathcal {D}})$. Since $S_h$ is finite dimensional all notions for ${\mathbb {R}}^d$-valued random variables carry over to this case in a straight-forward way. However, we often use the norm of the Bochner space $L^p(\varOmega ;V)$ with either $V = H^1_0({\mathcal {D}})$ or $V=L^2({\mathcal {D}})$, which is given by

$$\begin{aligned} \Vert X \Vert _{L^p(\varOmega ;V)} = \big ( {\mathbb {E}}\big [ \Vert X\Vert ^p_V \big ] \big )^{\frac{1}{p}} = \Big ( \int _{\varOmega }\Vert X(\omega )\Vert ^p_V \,\textrm{d}{\mathbb {P}}(\omega ) \Big )^{\frac{1}{p}} \end{aligned}$$

for $p \in [1,\infty )$. For an introduction to Bochner spaces we refer to [7, Appendix E].

## A randomized quadrature formula on a triangulation

As already mentioned in the introduction, quadrature rules are often used for the assembly of the matrix-vector system (1.4) associated to the finite element method for (2.12). In this section, inspired by the stratified Monte Carlo method, we introduce the first randomized quadrature formula, which is linked to the underlying triangulation $\mathcal {T}_h$ of the finite element space $S_h$. We discuss the well-posedness of the resulting method and derive error estimates in a similar way as for deterministic quadrature rules shown in [28, Section 5.6].

### An unbiased randomized quadrature

Let $\mathcal {T}_h$, $h \in (0,1]$, be an admissible triangulation of ${\mathcal {D}}$. For a given $v \in L^1({\mathcal {D}})$, we consider the following Monte Carlo estimator

3.19 $$\begin{aligned} Q_{\text {MC}}[v] := \sum _{T \in \mathcal {T}_h} |T| v( Z_T), \end{aligned}$$

where we sum over all triangles of the triangulation $\mathcal {T}_h$. Hereby, $(Z_T)_{T \in \mathcal {T}_h}$ denotes an independent family of random variables such that for each triangle $T \in \mathcal {T}_h$ the random variable $Z_T$ is uniformly distributed on *T*, that is $Z_T \sim \mathcal {U}(T)$. We discuss the simulation of $Z_T$ and the implementation of $Q_{\text {MC}}$ in Subsection 5.4.

Observe that the randomized quadrature rule is independent of the considered equivalence class of $v \in L^1({\mathcal {D}})$. If $v(x) = \tilde{v}(x)$ for almost every $x \in {\mathcal {D}}$, then it follows that $Q_{\text {MC}}[v] = Q_{\text {MC}}[\tilde{v}]$ with probability one.

#### Lemma 1

Let $\mathcal {T}_h$ be an admissible triangulation with maximal edge length $h \in (0,1]$. Then, the randomized quadrature rule $Q_{\text {MC}}$ is unbiased, i.e., for every $v \in L^1({\mathcal {D}})$ it holds

$$\begin{aligned} {\mathbb {E}}\big [ Q_{\text {MC}}[v] \big ] = \int _{\mathcal {D}}v(x) \,\textrm{d}x. \end{aligned}$$

Moreover, if $v \in L^2({\mathcal {D}})$ then it holds that

$$\begin{aligned} {\mathbb {E}}\Big [ \Big | \int _{\mathcal {D}}v(x) \,\textrm{d}x - Q_{\text {MC}}[v] \Big |^2 \Big ] \le \frac{\sqrt{3}}{2} h^2 \Vert v \Vert _{L^2({\mathcal {D}})}^2. \end{aligned}$$

In addition, if $v \in W^{s,2}({\mathcal {D}})$ for some $s \in (0,1)$ then it follows that

$$\begin{aligned} {\mathbb {E}}\Big [ \Big | \int _{\mathcal {D}}v(x) \,\textrm{d}x - Q_{\text {MC}}[v] \Big |^2 \Big ] \le h^{2 + 2s} | v |_{W^{s,2}({\mathcal {D}})}^2. \end{aligned}$$

#### Proof

Due to $Z_T \sim \frac{1}{|T|} \mathbb {I}_T(z) \,\textrm{d}z$ for every $T \in \mathcal {T}_h$ we have

$$\begin{aligned} {\mathbb {E}}\big [ |T| v(Z_T) \big ] = |T| \int _{\mathcal {D}}v(z) \frac{1}{|T|} \mathbb {I}_T(z) \,\textrm{d}z= \int _T v(z) \,\textrm{d}z. \end{aligned}$$

Then the first assertion follows by summing over all triangles of the triangulation.

Now, let $v \in L^2({\mathcal {D}})$ be arbitrary. Then, the mean-square error is equal to

$$\begin{aligned} {\mathbb {E}}\Big [ \Big | \int _{\mathcal {D}}v(x) \,\textrm{d}x - Q_{\text {MC}}[v] \Big |^2 \Big ]&= {\mathbb {E}}\Big [ \Big | \sum _{T \in \mathcal {T}_h} \Big ( \int _T v(x) \,\textrm{d}x - |T| v(Z_T) \Big ) \Big |^2 \Big ]\\&= \sum _{T \in \mathcal {T}_h} {\mathbb {E}}\Big [ \Big | \int _T v(x) \,\textrm{d}x - |T| v(Z_T) \Big |^2 \Big ] \end{aligned}$$

since the summands are independent and centered random variables. Therefore, they are orthogonal with respect to the $L^2(\varOmega )$-inner product as can easily be deduced from (2.18).

Next, for every $T \in \mathcal {T}_h$ we make use of $Z_T \sim \frac{1}{|T|} \mathbb {I}_T(z) \,\textrm{d}z$ and the Cauchy–Schwarz inequality. This yields

3.20 $$\begin{aligned} {\mathbb {E}}\Big [ \Big | \int _T v(x) \,\textrm{d}x - |T| v(Z_T) \Big |^2 \Big ]&= |T|^2 {\mathbb {E}}\Big [ \Big | \frac{1}{|T|} \int _T v(x) \,\textrm{d}x - v(Z_T) \Big |^2 \Big ] \nonumber \\&= |T| \int _{T} \Big | \frac{1}{|T|} \int _T v(x) \,\textrm{d}x - v(z) \Big |^2 \,\textrm{d}z\\&\le \int _{T} \int _{T} \big | v(x) - v(z) \big |^2 \,\textrm{d}x \,\textrm{d}z. \nonumber \end{aligned}$$

Then, since $v \in L^2({\mathcal {D}})$ we get

$$\begin{aligned} \int _{T} \int _{T} \big | v(x) - v(z) \big |^2 \,\textrm{d}x \,\textrm{d}z&= \int _T \int _T \big ( v(x)^2 - 2 v(x) v(z) + v(z)^2 \big ) \,\textrm{d}x \,\textrm{d}z\\&= 2 |T| \int _{T} \big | v(x) \big |^2 \,\textrm{d}x - 2 \Big (\int _T v(x) \,\textrm{d}x \Big )^2\\&\le 2 |T| \int _{T} \big | v(x) \big |^2 \,\textrm{d}x. \end{aligned}$$

Then, we recall Weitzenböck’s inequality [38], which yields an upper bound for the area |*T*| of a triangle $T \in \mathcal {T}_h$ with maximal edge length *h*. More precisely, it holds

3.21 $$\begin{aligned} |T| \le \frac{\sqrt{3}}{4} h^2. \end{aligned}$$

Hence, after summing over all triangles we obtain

$$\begin{aligned} \Big \Vert \int _{\mathcal {D}}v(x) \,\textrm{d}x - Q_{\text {MC}}[v] \Big \Vert _{L^2(\varOmega )}^2 \le 2 \sum _{T \in \mathcal {T}_h} |T| \int _{T} \big | v(x) \big |^2 \,\textrm{d}x \le \frac{\sqrt{3}}{2} h^2 \Vert v \Vert ^2_{L^2({\mathcal {D}})}. \end{aligned}$$

This proves the second claim.

Finally, let $v \in W^{s,2}({\mathcal {D}})$, $s \in (0,1)$. The estimate in (3.20) is then continued by

$$\begin{aligned} {\mathbb {E}}\Big [ \Big | \int _T v(x) \,\textrm{d}x - |T| v(Z_T) \Big |^2 \Big ]&\le \int _{T} \int _{T} \big | v(x) - v(z) \big |^2 \,\textrm{d}x \,\textrm{d}z\\&\le h^{2 + 2s} \int _{T} \int _{T} \frac{ \big | v(x) - v(z) \big |^2}{|x - z|^{2 + 2 s}} \,\textrm{d}x \,\textrm{d}z\\&= h^{2 + 2s} | v |_{W^{s,2}(T)}^2 \end{aligned}$$

since $|x - z| \le h$ for all $x,y \in T$. After summing over all triangles we directly obtain the third assertion. $\square$

### Integrating the randomized quadrature into FEM

Next, we apply the randomized quadrature formula (3.19) for the approximation of the bilinear form *a* and the linear form *F* defined in (2.8) and (2.9). From this we obtain two randomized mappings $a_{\text {MC}} :S_h \times S_h \rightarrow L^\infty (\varOmega )$ and $F_{\text {MC}} :S_h \rightarrow L^2(\varOmega )$ which are given by

3.22 $$\begin{aligned} a_{\text {MC}}(v_h,w_h) := Q_{\text {MC}}[\sigma \nabla v_h \cdot \nabla w_h] = \sum _{T \in \mathcal {T}_h} |T| \sigma (Z_T) \nabla v_h(Z_T) \cdot \nabla w_h(Z_T) \end{aligned}$$

and

3.23 $$\begin{aligned} F_{\text {MC}}(v_h) := Q_{\text {MC}}[f v_h] = \sum _{T \in \mathcal {T}_h} |T| f(Z_T) v_h(Z_T) \end{aligned}$$

for all $v_h, w_h \in S_h$. In passing, we observe that $a_{\text {MC}}(v_h,w_h) = a(v_h,w_h)$ if $\sigma \equiv c \in ( 0,\infty )$ in ${\mathcal {D}}$. This holds true since the gradients of $v_h, w_h \in S_h$ are constant on each triangle.

The next lemma answers the question of well-posedness of $a_{\text {MC}}$ and $F_{\text {MC}}$ and contains some additional properties.

#### Lemma 2

Let $(\mathcal {T}_h)_{h \in (0,1]}$ be a family of admissible triangulations of ${\mathcal {D}}$. Assume that $\sigma \in L^\infty ({\mathcal {D}})$ satisfies $\sigma (x) \ge \sigma _0 > 0$ for almost every $x \in {\mathcal {D}}$. Then, the mapping $a_{\text {MC}}$ introduced in (3.22) is well-defined for every $h \in (0,1]$. Moreover, it holds ${\mathbb {P}}$-almost surely that

$$\begin{aligned} |a_{\text {MC}}(v_h,w_h)|&\le \Vert \sigma \Vert _{L^\infty ({\mathcal {D}})} | v_h |_{H^1({\mathcal {D}})} |w_h|_{H^1({\mathcal {D}})},\\ a_{\text {MC}}(v_h, v_h)&\ge \sigma _0 |v_h|_{H^1({\mathcal {D}})}^2 \end{aligned}$$

for all $$v_h, w_h \in S_h$$.

In addition, if $f \in L^2({\mathcal {D}})$ and the family of triangulations satisfies Assumption 2 then the mapping $F_{\text {MC}}$ defined in (3.23) is also well-defined and there exists $C \in (0,\infty )$ independent of $\mathcal {T}_h$ with

$$\begin{aligned} |F_{\text {MC}}(v_h)|&\le C \ell _h^{\frac{1}{2}} Q_{\text {MC}}[|f|] | v_h |_{H^1({\mathcal {D}})} < \infty \quad {\mathbb {P}}\text { -a.s.},\\ \Vert F_{\text {MC}}(v_h) \Vert _{L^2(\varOmega )}&\le C \Vert f \Vert _{L^2({\mathcal {D}})} |v_h|_{H^1({\mathcal {D}})} \end{aligned}$$

for all $v_h \in S_h$, where $\ell _h = \max ( 1, \log (1/h))$.

#### Proof

We first show that $a_{\text {MC}}(v_h, w_h) \in L^\infty (\varOmega )$ for every $v_h, w_h \in S_h$. To see this, we recall that the functions in $S_h$ are linear on each triangle *T* in $\mathcal {T}_h$. This implies that the gradient $\nabla v_h$ is piecewise constant for every $v_h \in S_h$. Hence, the random variables $\nabla v_h(Z_T)$, $T \in \mathcal {T}_h$, are, in fact, constant with probability one. This implies that

$$\begin{aligned} |T| |\nabla v_h(Z_T)|^2 = \int _T |\nabla v_h(x)|^2 \,\textrm{d}x\quad {\mathbb {P}}\text { -almost surely.} \end{aligned}$$

Together with the assumption $\sigma \in L^\infty ({\mathcal {D}})$ it therefore follows that the summands in (3.22) are essentially bounded random variables. More precisely, it holds ${\mathbb {P}}$-almost surely that

$$\begin{aligned} |a_{\text {MC}}(v_h, w_h)|&\le \sum _{T \in \mathcal {T}_h} |T| \sigma (Z_T) | \nabla v_h(Z_T)| |\nabla w_h(Z_T)| \\&\le \Vert \sigma \Vert _{L^\infty ({\mathcal {D}})} \sum _{T \in \mathcal {T}_h} |T| | \nabla v_h(Z_T)| |\nabla w_h(Z_T)|\\&\le \Vert \sigma \Vert _{L^\infty ({\mathcal {D}})} \Big ( \sum _{T \in \mathcal {T}_h} |T| | \nabla v_h(Z_T)|^2 \Big )^{\frac{1}{2}} \Big ( \sum _{T \in \mathcal {T}_h} |T| | \nabla w_h(Z_T)|^2 \Big )^{\frac{1}{2}}\\&= \Vert \sigma \Vert _{L^\infty ({\mathcal {D}})} |v_h|_{H^1({\mathcal {D}})} |w_h|_{H^1({\mathcal {D}})} \end{aligned}$$

for all $$v_h, w_h \in S_h$$.

Moreover, the same arguments yield for every $v_h \in S_h$

$$\begin{aligned} a_{\text {MC}}(v_h, v_h) = \sum _{T \in \mathcal {T}_h} |T| \sigma (Z_T) | \nabla v_h(Z_T)|^2 \ge \sigma _0 |v_h|^2_{H^1({\mathcal {D}})} \quad {\mathbb {P}}\text {-almost surely,} \end{aligned}$$

since $\sigma (Z_T) \ge \sigma _0 > 0$ almost surely.

Next, we turn to the mapping $F_{\text {MC}}$. From (2.13) it follows for $v_h \in S_h$ that

$$\begin{aligned} |F_{\text {MC}}(v_h)|&\le \sum _{T \in \mathcal {T}_h} |T| |f(Z_T)| |v_h(Z_T)| \le \Vert v_h \Vert _{L^\infty ({\mathcal {D}})} Q_{\text {MC}}[|f|]\\&\le C \ell _h^{\frac{1}{2}} Q_{\text {MC}}[|f|] | v_h |_{H^1({\mathcal {D}})}. \end{aligned}$$

Observe that the bound on the right-hand side still contains a random quadrature formula and is, therefore, itself random. However, for $f \in L^2({\mathcal {D}})$ it follows from applications of the Cauchy–Schwarz inequality and Lemma 1 that

$$\begin{aligned} {\mathbb {E}}\big [ ( Q_{\text {MC}}[|f|])^2 \big ]&= {\mathbb {E}}\Big [ \Big ( \sum _{T \in \mathcal {T}_h} |T| |f(Z_T)| \Big )^2 \Big ]\\&\le |{\mathcal {D}}| {\mathbb {E}}\Big [ \sum _{T \in \mathcal {T}_h} |T| |f(Z_T)|^2 \Big ]\\&= |{\mathcal {D}}| \int _{\mathcal {D}}|f(z)|^2 \,\textrm{d}z. \end{aligned}$$

In particular, we have that $Q_{\text {MC}}[|f|] < \infty$ with probability one. This also proves that $F_{\text {MC}}(v_h) \in L^2(\varOmega )$. It remains to prove the asserted estimate of the $L^2(\varOmega )$-norm of $F_{\text {MC}}(v_h)$. For this we first observe that

$$\begin{aligned} \Vert F_{\text {MC}}(v_h) \Vert ^2_{L^2(\varOmega )} = \big \Vert F_{\text {MC}}(v_h) - {\mathbb {E}}[ F_{\text {MC}}(v_h) ] \big \Vert ^2_{L^2(\varOmega )} + \big ( {\mathbb {E}}[ F_{\text {MC}}(v_h) ] \big )^2 \end{aligned}$$

for every $v_h \in S_h$. From Lemma 1, the Cauchy–Schwarz inequality, and the Poincaré inequality on $H^1_0({\mathcal {D}})$ it follows that

$$\begin{aligned} \big ( {\mathbb {E}}[ F_{\text {MC}}(v_h)] \big )^2&= \Big ( \int _{{\mathcal {D}}} f(x) v_h(x) \,\textrm{d}x \Big )^2\\&\le \int _{{\mathcal {D}}} |f(x)|^2 \,\textrm{d}x \int _{{\mathcal {D}}} |v_h(x)|^2 \,\textrm{d}x \le C \Vert f \Vert _{L^2({\mathcal {D}})}^2 | v_h |_{H^1({\mathcal {D}})}^2, \end{aligned}$$

where the constant *C* only depends on ${\mathcal {D}}$. An application of Lemma 1 then yields

$$\begin{aligned} {\mathbb {E}}\big [ \big | F_{\text {MC}}(v_h) - {\mathbb {E}}[ F_{\text {MC}}(v_h) ] \big |^2 \big ]&= {\mathbb {E}}\Big [ \Big | Q_{\text {MC}}[f v_h] - \int _{\mathcal {D}}f(x) v_h(x) \,\textrm{d}x \Big |^2 \Big ]\\&\le \frac{\sqrt{3}}{2} h^2 \Vert f v_h \Vert _{L^2({\mathcal {D}})}^2\\&\le \frac{\sqrt{3}}{2} h^2 \Vert f \Vert _{L^2({\mathcal {D}})}^2 \Vert v_h\Vert _{L^\infty ({\mathcal {D}})}^2\\&\le C \frac{\sqrt{3}}{2} h^2 \ell _h \Vert f \Vert _{L^2({\mathcal {D}})}^2 |v_h|_{H^1(\varOmega )}^2, \end{aligned}$$

where we also applied the maximum norm estimate (2.13). Hence, after taking note of $\sup _{h \in (0,1]} h^2 \ell _h = \sup _{h \in (0,1]} h^2 \max (1,\log (1/h)) < \infty$ the proof is completed. $\square$

Next, we introduce the finite element problem based on the randomized quadrature rule. In terms of $a_{\text {MC}}$ and $F_{\text {MC}}$ the problem is stated as follows:

3.24 $$\begin{aligned} {\left\{ \begin{array}{ll} \text {Find } u_h^{\text {MC}} :\varOmega \rightarrow S_h \text { such that } {\mathbb {P}}\text {-almost surely} \\ a_{\text {MC}}(u_h^{\text {MC}},v_h) = F_{\text {MC}}(v_h) \text { for all } v_h \in S_h. \end{array}\right. } \end{aligned}$$

#### Theorem 1

Suppose that $f \in L^2({\mathcal {D}})$ and $\sigma \in L^\infty ({\mathcal {D}})$ with $\sigma (x) \ge \sigma _0 > 0$ for almost every $x \in {\mathcal {D}}$ are given. Then, for every admissible triangulation $\mathcal {T}_h$, $h \in (0,1]$, there exists a uniquely determined solution $u_h^{\text {MC}} :\varOmega \rightarrow S_h$ to the discrete problem (3.24). In addition, there exists $C \in (0,\infty )$ independent of $\mathcal {T}_h$ such that

$$\begin{aligned} |u_h^{\text {MC}}|_{H^1({\mathcal {D}})}&\le C \ell _h^{\frac{1}{2}} Q_{\text {MC}}[|f|] \quad {\mathbb {P}}\text {-a.s.,} \end{aligned}$$

where $$\ell _h = \max (1,\log (1/h))$$.

#### Proof

It follows from Lemma 2 that the bilinear form $a_{\text {MC}}$ is ${\mathbb {P}}$-almost surely strictly positive and bounded. Moreover, an inspection of the proof reveals that the exceptional set $N_1 \subset \varOmega$ of probability zero, where these properties might be violated, can be chosen independently of the arguments $v_h, w_h \in S_h$. This is true since only the gradients of $v_h$ and $w_h$ appear in $a_{\text {MC}}(v_h,w_h)$, which are piecewise constant on each triangle. Hence, on the set $\{ Z_T \in T\} \in {\mathcal {F}}$, which has probability one, the randomness only occurs in the coefficient function $\sigma$. Therefore, for every $\omega \in \varOmega \setminus N_1$ the mapping $S_h \times S_h \ni (v_h,w_h) \mapsto a_{\text {MC}}(v_h,w_h)(\omega ) \in {\mathbb {R}}$ satisfies the conditions of the lemma of Lax–Milgram.

In the same way, there exists a measurable set $N_2 \subset \varOmega$ of probability zero such that the mapping $S_h \ni v_h \mapsto F_{\text {MC}}(v_h)(\omega ) \in {\mathbb {R}}$ is a bounded linear functional on $H^1_0({\mathcal {D}})$ for all $\omega \in \varOmega \setminus N_2$. In particular, we observe that the exceptional set $N_2$ can again be chosen independently of the mapping $v_h$ due to the continuity of all elements in $S_h$. In addition, the following estimate, which was used in the proof of Lemma 2, is true for all $\omega \in \varOmega$:

$$\begin{aligned} |v_h(Z_T(\omega ))| \le \Vert v_h \Vert _{L^\infty ({\mathcal {D}})}. \end{aligned}$$

Consequently, for every fixed $\omega \in \varOmega \setminus (N_1 \cup N_2)$ the lemma of Lax–Milgram uniquely determines an element $u_h^{\text {MC}}(\omega ) \in S_h$ satisfying

3.25 $$\begin{aligned} a_{\text {MC}}(u_h^{\text {MC}}(\omega ), v_h)(\omega ) = F_{\text {MC}}(v_h)(\omega ) \quad \text {for all } v_h \in S_h. \end{aligned}$$

Let us define $u_h^{\text {MC}}(\omega ) = 0 \in S_h$ for all $\omega \in N_1 \cup N_2$. Next, we have to prove that the mapping $\varOmega \ni \omega \mapsto u_h^{\text {MC}}(\omega ) \in S_h$ is measurable. However, this follows from an application of [10, Lemma 4.3] to the mapping $g :\varOmega \times {\mathbb {R}}^{N_h} \rightarrow {\mathbb {R}}^{N_h}$ defined by $g(v ,\omega ) := [ a_{\text {MC}}( \sum _{i = 1}^{N_h} v_i \psi _i, \psi _j)(\omega ) - F_{\text {MC}}(\psi _j)]_{j = 1}^{N_h}$, where $v = [v_i]_{i = 1}^{N_h} \in {\mathbb {R}}^{N_h}$ and $(\psi _j)_{j = 1}^{N_h} \subset S_h$ is an arbitrary basis of the finite dimensional space $S_h$.

It remains to prove the stability estimate. Due to Lemma 2 and (3.25) it holds on $\varOmega \setminus (N_1 \cup N_2)$ that

$$\begin{aligned} \sigma _0 |u_h^{\text {MC}}|^2_{H^1({\mathcal {D}})} \le a_{\text {MC}}(u_h^{\text {MC}},u_h^{\text {MC}}) = F_{\text {MC}}(u_h^{\text {MC}}) \le C \ell _h^{\frac{1}{2}} Q_{\text {MC}}[|f|] |u_h^{\text {MC}}|_{H^1({\mathcal {D}})}. \end{aligned}$$

Hence, after canceling the norm of $u_h^{\text {MC}}$ one time on both sides of the inequality we obtain the desired estimate. $\square$

Let us emphasize that the solution to the discrete problem (3.24) is a random variable. In fact, it follows directly from Theorem 1 that $u_h^{\text {MC}} \in L^p(\varOmega ;H^1_0({\mathcal {D}}))$ provided $f \in L^p({\mathcal {D}})$ for $p \in [2,\infty ]$.

As in the standard error analysis (cf. [28, Theorem 5.7]), we want to use $u_h^{\text {MC}}$ as a test function in the discrete problem (3.24). However, in contrast to the situation in Lemma 1 we have, in general, that $|{\mathbb {E}}[ F_{\text {MC}}(v_h)] - F( {\mathbb {E}}[v_h] )| \ne 0$ for an arbitrary $S_h$-valued random function $v_h \in L^2(\varOmega ;H^1_0({\mathcal {D}}))$. The following lemmas give an estimate of this difference when *f* lives in different spaces.

#### Lemma 3

Let Assumption 2 be satisfied. Then, there exists $C \in (0,\infty )$ such that for every $h \in (0,1]$, $f \in L^p({\mathcal {D}})$, $p \in [2,\infty ]$, and $S_h$-valued random variable $v_h \in L^2(\varOmega ;H^1_0({\mathcal {D}}))$ it holds

$$\begin{aligned} \big | {\mathbb {E}}\big [ F_{MC}(v_h) - F(v_h) \big ] \big | \le {\left\{ \begin{array}{ll} C h^{1 - \frac{2}{p}} \Vert f\Vert _{L^p({\mathcal {D}})} \Vert v_h \Vert _{L^2(\varOmega ;H^1_0({\mathcal {D}}))}, & \quad \text { if } p \in [2,\infty ),\\ C \ell _h^{\frac{1}{2}} h \Vert f\Vert _{L^\infty ({\mathcal {D}})} \Vert v_h \Vert _{L^2(\varOmega ;H^1_0({\mathcal {D}}))}, & \quad \text { if } p = \infty , \end{array}\right. } \end{aligned}$$

where $$\ell _h = \max (1,\log (1/h))$$.

#### Proof

For the error analysis it is convenient to choose an $L^2({\mathcal {D}})$-orthonormal basis $(\psi _j)_{j = 1}^{N_h}$ of $S_h$, which solves the discrete eigenvalue problem

3.26 $$\begin{aligned} a(\psi _j, w_h) = \lambda _{h,j} (\psi _j, w_h)_{L^2({\mathcal {D}})} \end{aligned}$$

for all $w_h \in S_h$. Hereby, $0 < \lambda _{h,1} \le \lambda _{h,2} \le \ldots \le \lambda _{h,N_h}$ denote the discrete eigenvalues of the bilinear form *a* on the finite element space $S_h \subset H^{1}_0({\mathcal {D}})$. We refer to [28, Section 6.2] regarding the existence of $(\lambda _{h,j})_{j = 1}^{N_h}$ and the associated orthonormal basis $(\psi _j)_{j = 1}^{N_h}$.

Next, let $h \in (0,1]$, $f \in L^p({\mathcal {D}})$, $p \in [2,\infty ]$, and an $S_h$-valued random variable $v_h \in L^2(\varOmega ;H^1_0({\mathcal {D}}))$ be arbitrary. Then, we represent $v_h$ in terms of the orthonormal basis $(\psi _j)_{j = 1}^{N_h} \subset S_h$ by

3.27 $$\begin{aligned} v_h = \sum _{j = 1}^{N_h} v_j \psi _j, \end{aligned}$$

For this choice of the basis, the random coefficients $(v_j)_{j = 1}^{N_h} \subset L^2(\varOmega )$ are given by

$$\begin{aligned} v_j = ( v_h, \psi _j)_{L^2({\mathcal {D}})}. \end{aligned}$$

In particular, it follows from the Cauchy–Schwarz inequality that $v_j$ is indeed a real-valued and square-integrable random variable for every $j \in \{1,\ldots ,N_h\}$. Due to the linearity of *F* and $F_{MC}$ we then arrive at the estimate

$$\begin{aligned}&\big | {\mathbb {E}}\big [ F_{MC}(v_h) - F(v_h) \big ] \big | \\&\quad = \Big | \sum _{j = 1}^{N_h} {\mathbb {E}}\big [ v_j \big ( F_{MC}(\psi _j) - F(\psi _j)\big ) \big ] \Big |\\&\quad \le \sum _{j = 1}^{N_h} \big ( {\mathbb {E}}\big [ |v_j |^2 \big ] \big )^{\frac{1}{2}} \big ( {\mathbb {E}}\big [ \big | F_{MC}(\psi _j) - F(\psi _j)\big |^2 \big ] \big )^{\frac{1}{2}}\\&\quad \le \Big ( \sum _{j = 1}^{N_h} \lambda _{h,j} {\mathbb {E}}\big [ |v_j |^2 \big ] \Big )^{\frac{1}{2}} \Big ( \sum _{j = 1}^{N_h} \lambda _{h,j}^{-1} {\mathbb {E}}\big [ \big | F_{MC}(\psi _j) - F(\psi _j)\big |^2 \big ] \Big )^{\frac{1}{2}} \end{aligned}$$

by additional applications of the Cauchy–Schwarz inequality. From (3.27) and (3.26) we then get

$$\begin{aligned} a (v_h, v_h) = \sum _{i,j=1}^{N_h} v_j v_i a(\psi _j,\psi _i) = \sum _{i,j=1}^{N_h} \lambda _{h,j} v_i v_j (\psi _j,\psi _i)_{L^2({\mathcal {D}})} = \sum _{j=1}^{N_h} \lambda _{h,j} v_j^2, \end{aligned}$$

since $(\psi _{j})_{j = 1}^{N_h}$ is an orthonormal basis of $S_h$. From this it follows that

3.28 $$\begin{aligned} \Big ( \sum _{j = 1}^{N_h} \lambda _{h,j} {\mathbb {E}}\big [ |v_j |^2 \big ] \Big )^{\frac{1}{2}} = \big ( {\mathbb {E}}\big [ a(v_h,v_h) \big ] \big )^\frac{1}{2} \le \Vert \sigma \Vert _{L^\infty ({\mathcal {D}})}^{\frac{1}{2}} \Vert v_h \Vert _{L^2(\varOmega ;H^1_0({\mathcal {D}}))}. \end{aligned}$$

Moreover, an application of Lemma 1 yields

$$\begin{aligned} {\mathbb {E}}\big [ \big | F_{MC}(\psi _j) - F(\psi _j)\big |^2 \big ] = {\mathbb {E}}\Big [ \Big | Q_{MC}(f \psi _j) - \int _{\mathcal {D}}f \psi _j \,\textrm{d}x \Big |^2 \Big ] \le \frac{\sqrt{3}}{2} h^2 \Vert f \psi _j\Vert _{L^2({\mathcal {D}})}^2 \end{aligned}$$

for every $j \in \{1,\ldots ,N_h\}$. Further, since $f \in L^p({\mathcal {D}})$, $p \in [2,\infty ]$, it follows from an application of Hölder’s inequality with conjugated exponent $p' \in [2,\infty ]$ determined by $\frac{1}{p} + \frac{1}{p'} = \frac{1}{2}$ that

$$\begin{aligned} \Vert f \psi _j\Vert _{L^2({\mathcal {D}})} \le \Vert f \Vert _{L^p({\mathcal {D}})} \Vert \psi _j\Vert _{L^{p'}({\mathcal {D}})}. \end{aligned}$$

An application of the Gagliardo–Nierenberg inequality, cf. [33, Theorem 1.24], yields

$$\begin{aligned} \Vert \psi _j \Vert _{L^{p'}({\mathcal {D}})} \le C \Vert \psi _j \Vert _{L^2({\mathcal {D}})}^{\frac{2}{p'}} | \psi _j|_{H^1({\mathcal {D}})}^{1 - \frac{2}{p'}}, \end{aligned}$$

where the constant *C* is independent of $j \in \{1,\ldots ,N_h\}$. Since $\Vert \psi _j \Vert _{L^2({\mathcal {D}})} = 1$ for every $j \in \{1,\ldots ,N_h\}$ and due to (2.10) and (3.26) we therefore obtain

$$\begin{aligned} \Vert \psi _j \Vert _{L^{p'}({\mathcal {D}})} \le C | \psi _j|_{H^1({\mathcal {D}})}^{\frac{2}{p}} \le \frac{C}{\sigma _0^{\frac{1}{p}}} a(\psi _j,\psi _j)^{\frac{1}{p}} \le \frac{C}{\sigma _0^{\frac{1}{p}}} \lambda _{h,j}^{\frac{1}{p}} \end{aligned}$$

for every $p, p' \in [2,\infty ]$ with $\frac{1}{p} + \frac{1}{p'} = \frac{1}{2}$. Altogether, we have the bound

3.29 $$\begin{aligned} \begin{aligned} \sum _{j = 1}^{N_h} \lambda _{h,j}^{-1} {\mathbb {E}}\big [ \big | F_{MC}(\psi _j) - F(\psi _j)\big |^2 \big ]&\le \frac{\sqrt{3}}{2} h^2 \Vert f \Vert _{L^p({\mathcal {D}})}^2 \sum _{j = 1}^{N_h} \lambda _{h,j}^{-1} \Vert \psi _j \Vert _{L^{p'}({\mathcal {D}})}^2 \\&\le C h^{2} \Vert f \Vert _{L^p({\mathcal {D}})}^2 \sum _{j = 1}^{N_h} \lambda _{h,j}^{-1 + \frac{2}{p}}. \end{aligned} \end{aligned}$$

Concerning the last sum we recall from [28, Theorem 6.7] that

$$\begin{aligned} \lambda _{j} \le \lambda _{h,j} \end{aligned}$$

for all $j \in \{1,\ldots ,N_h\}$, where $(\lambda _j)_{j \in {\mathbb {N}}}$ denotes the family of eigenvalues of the bilinear form *a* on the full space $H^1_0({\mathcal {D}})$. Moreover, it is well-known, cf. [28, Section 6.1], that there exist constants $c_1, c_2 \in (0,\infty )$ only depending on $\sigma$ and ${\mathcal {D}}$ such that

$$\begin{aligned} c_1 j \le \lambda _j \le c_2 j. \end{aligned}$$

From this it follows that

$$\begin{aligned} \sum _{j = 1}^{N_h} \lambda _{h,j}^{-1+ \frac{2}{p}}&\le \sum _{j = 1}^{N_h} \lambda _{j}^{-1+ \frac{2}{p}} \le c_1^{-1 + \frac{2}{p}} \sum _{j = 1}^{N_h} j^{-1+ \frac{2}{p}} \le c_1^{-1 + \frac{2}{p}} \Big (1 + \int _{1}^{N_h} y^{-1 + \frac{2}{p}} \,\textrm{d}y \Big ). \end{aligned}$$

Hence, we obtain

$$\begin{aligned} \sum _{j = 1}^{N_h} \lambda _{h,j}^{-1+ \frac{2}{p}} \le {\left\{ \begin{array}{ll} \frac{p}{2} c_1^{-1 + \frac{2}{p}} N_h^{\frac{2}{p}}, & \quad \text {if } p \in [2,\infty ),\\ c_1^{-1} (1 + \log (N_h)), & \quad \text {if } p = \infty . \end{array}\right. } \end{aligned}$$

From (3.26), (2.11), and the inverse estimate (2.14) it then follows that

$$\begin{aligned} N_h \le \frac{1}{c_1} \lambda _{h,N_h} = \frac{1}{c_1} a(\psi _{N_h},\psi _{N_h}) \le \frac{1}{c_1} \Vert \sigma \Vert _{L^\infty ({\mathcal {D}})} | \psi _{N_h}|^2_{H^1({\mathcal {D}})} \le C h^{-2}. \end{aligned}$$

This implies that $\log (N_h) \le C \max (1, \log (1/h)) = C \ell _h$. Altogether, this yields

3.30 $$\begin{aligned} \sum _{j = 1}^{N_h} \lambda _{h,j}^{-1 + \frac{2}{p}} \le {\left\{ \begin{array}{ll} C h^{- \frac{4}{p}},& \quad \text {if } p \in [2,\infty ),\\ C \ell _h,& \quad \text {if } p = \infty . \end{array}\right. } \end{aligned}$$

Combining this with (3.28) and (3.29) then completes the proof. $\square$

#### Lemma 4

Let Assumption 2 be satisfied. Then there exists $C \in (0,\infty )$ such that for every $h \in (0,1]$, $f \in W^{s',2}({\mathcal {D}})$, $s'\in (0,1)$, and $S_h$-valued random variable $v_h \in L^2(\varOmega ;H^1_0({\mathcal {D}}))$ it holds

$$\begin{aligned} \big | {\mathbb {E}}\big [ F_{\text {MC}}(v_h) - F(v_h) \big ] \big | \le {Ch^{s'}\Vert f\Vert _{W^{s',2}({\mathcal {D}})} \Vert v_h \Vert _{L^2(\varOmega ;H^1_0({\mathcal {D}}))}.} \end{aligned}$$

#### Proof

To show the quadrature estimate, we may need to introduce some auxiliary quadrature

3.31 $$\begin{aligned} F_{\text {sMC}}(v_h) := \sum _{T \in \mathcal {T}_h} |T| f(Z_T) v_h(C_T) \end{aligned}$$

for all $v_h\in S_h$ and $C_T$ is the centroid of the triangle of *T*. Note that

3.32 $$\begin{aligned} \int _T v_h(x) \,\textrm{d}x=|T|v_h (C_T) \end{aligned}$$

because of $v_h$ being piecewise linear on every *T*, see [28, Eq. (5.61)].

Now we may decompose our target term as

$$\begin{aligned}&\big | {\mathbb {E}}\big [ F_{\text {MC}}(v_h) - F(v_h) \big ] \big | \\&\quad \le \big | {\mathbb {E}}\big [ F_{\text {MC}}(v_h) - F_{\text {sMC}}(v_h) \big ] \big |\\&\qquad +\big | {\mathbb {E}}\big [ F_{\text {sMC}}(v_h) - \sum _{T \in \mathcal {T}_h} \int _T f(x)\,v_h(C_T) \,\textrm{d}x\big ] \big |\\&\qquad +\big | {\mathbb {E}}\big [ \sum _{T \in \mathcal {T}_h} \int _T f(x)\,v_h(C_T) \,\textrm{d}x - F(v_h)\big ]\big |:=\sum _{i=1}^3 T_i, \end{aligned}$$

For $T_1$, we have

$$\begin{aligned}&\big | {\mathbb {E}}\big [ F_{\text {MC}}(v_h) - F_{\text {sMC}}(v_h) \big ] \big |\\&\quad = \Big | {\mathbb {E}}\Big [ \sum _{T \in \mathcal {T}_h} |T|f(Z_T)(v_h(Z_T)-v_h(C_T))\Big ] \Big |\\&\quad \le \sum _{T \in \mathcal {T}_h}\big ({\mathbb {E}}[|T||f(Z_T)|^2]\big )^{1/2} \big ({\mathbb {E}}[|T||v_h(Z_T)-v_h(C_T)|^2]\big )^{1/2}\\&\quad \le \Big (\sum _{T \in \mathcal {T}_h}{\mathbb {E}}[|T||f(Z_T)|^2]\Big )^{1/2} \Big (\sum _{T \in \mathcal {T}_h}{\mathbb {E}}[|T||v_h(Z_T)-v_h(C_T)|^2]\Big )^{1/2}\\&\quad \le \Vert f\Vert _{L^2(\mathcal {D})}\Big (h^2\sum _{T \in \mathcal {T}_h}{\mathbb {E}}[|T||\nabla v_h(C_T)|^2]\Big )^{1/2}\\&\quad =h \Vert f\Vert ^2_{L^2(\mathcal {D})}\Big (\sum _{T \in \mathcal {T}_h}{\mathbb {E}}\big [\int _T |\nabla v_h(x)|^2 \, \textrm{d}x\big ]\Big )^{1/2}\\&\quad =h \Vert f\Vert _{L^2(\mathcal {D})} \Vert v_h \Vert _{L^2(\varOmega ;H^1_0({\mathcal {D}}))}, \end{aligned}$$

where we use the fact that $\nabla v_h(\cdot )$ is constant on each *T* to derive the last second line. The idea to bound $T_3$ is similar,

$$\begin{aligned}&\big | {\mathbb {E}}\big [ \sum _{T \in \mathcal {T}_h} \int _T f(x)\,v_h(C_T) \,\textrm{d}x\big ] - F(v_h)\big |\\&\quad = \big | {\mathbb {E}}\big [ \sum _{T \in \mathcal {T}_h} \int _T f(x)\,(v_h(C_T)- v_h(x)\,\textrm{d}x\big ] \big |\\&\quad \le \Vert f\Vert _{L^2(\mathcal {D})}\Big (\sum _{T \in \mathcal {T}_h}{\mathbb {E}}[|T||v_h(C_T)-v_h(x)|^2]\Big )^{1/2}\\&\quad \le h \Vert f\Vert _{L^2(\mathcal {D})} \Vert v_h \Vert _{L^2(\varOmega ;H^1_0({\mathcal {D}}))}. \end{aligned}$$

For $T_2$, we will use the fact (3.32) and the Cauchy–Schwarz inequality to derive

$$\begin{aligned}&\big | {\mathbb {E}}\big [ F_{\text {sMC}}(v_h) - \sum _{T \in \mathcal {T}_h} \int _T f(x)\,v_h(C_T) \,\textrm{d}x\big ] \big |\\&= \Big | {\mathbb {E}}\Big [ \sum _{T \in \mathcal {T}_h} \int _T \big ( f(Z_T) - f(x) \big ) \, v_h(C_T) \,\textrm{d}x\Big ] \Big |\\&\le \Big | {\mathbb {E}}\Big [ \sum _{T \in \mathcal {T}_h} \Big (\int _T|f(Z_T)-f(x)|^2 \,\textrm{d}x\Big )^{1/2} \Big (\int _T\,|v_h(x)|^{2} \,\textrm{d}x\Big )^{1/{2}}\Big ] \Big |\\&\le \Big ({\mathbb {E}}\Big [ \sum _{T \in \mathcal {T}_h} \int _T|f(Z_T)-f(x)|^p \,\textrm{d}x\Big ]\Big )^{1/2} \Big ({\mathbb {E}}\Big [\sum _{T \in \mathcal {T}_h} \int _T\,|v_h(x)|^{2} \,\textrm{d}x\Big ] \Big )^{1/{2}} \\&= \Big ( \sum _{T \in \mathcal {T}_h} |T|^{-1}\int _T\int _T|f(z)-f(x)|^p \,\textrm{d}x\,\textrm{d}z\Big )^{1/2} \Vert v_h\Vert _{L^{2}(\varOmega ;L^{2}({\mathcal {D}}))} \\&\le \Big ( \sum _{T \in \mathcal {T}_h} |T|^{-1}h^{2+2s'}\int _T\int _T\frac{|f(z)-f(x)|^2}{|z-x|^{2+2s'}} \,\textrm{d}x\,\textrm{d}z\Big )^{1/2} \Vert v_h\Vert _{L^{2}(\varOmega ;L^{2}({\mathcal {D}}))} \\&\le h^s \Vert f\Vert _{W^{s',2}({\mathcal {D}})} \Vert v_h\Vert _{H^{1}_0(\varOmega ;L^{2}({\mathcal {D}}))}, \end{aligned}$$

where, to get the last second lines, we make use of fact that $|z-x|<h$ for all $z,x \in T$.

$$\square $$

Next, we state and prove the main result of this section.

#### Theorem 2

Suppose that $\sigma \in L^\infty ({\mathcal {D}}) \cap W^{s,q}({\mathcal {D}})$, $s \in (0,1]$, $q \in (2,\infty )$, with $\sigma (x) \ge \sigma _0 > 0$ for almost every $x \in {\mathcal {D}}$. Let Assumptions 1 and 2 be satisfied.

1. If $f \in L^p({\mathcal {D}})$, $p \in {(}2,\infty )$, then it holds
  $$\begin{aligned} \big \Vert u - u_h^{\text {MC}} \big \Vert _{L^2(\varOmega ;H^1_0({\mathcal {D}}))}&\le C h \Vert u \Vert _{H^2({\mathcal {D}})} + C h^s \Vert u \Vert _{H^2({\mathcal {D}})} | \sigma |_{W^{s,q}({\mathcal {D}})}\\&\quad + C h^{1 - \frac{2}{p}} \Vert f \Vert _{L^p({\mathcal {D}})} \end{aligned}$$
  for every $h \in (0,1]$. Further, if $f \in L^\infty ({\mathcal {D}})$ then it holds
  $$\begin{aligned} \big \Vert u - u_h^{\text {MC}} \big \Vert _{L^2(\varOmega ;H^1_0({\mathcal {D}}))}&\le C h \Vert u \Vert _{H^2({\mathcal {D}})} + C h^s \Vert u \Vert _{H^2({\mathcal {D}})} | \sigma |_{W^{s,q}({\mathcal {D}})}\\&\quad + C \ell _h^{\frac{1}{2}} h \Vert f \Vert _{L^\infty ({\mathcal {D}})}. \end{aligned}$$
2. Moreover, if $f\in W^{s',2}({\mathcal {D}})$, $s'\in (0,1)$, then
  $$\begin{aligned} \big \Vert u - u_h^{\text {MC}} \big \Vert _{L^2(\varOmega ;H^1_0({\mathcal {D}}))}&\le C h \Vert u \Vert _{H^2({\mathcal {D}})} + C h^s \Vert u \Vert _{H^2({\mathcal {D}})} | \sigma |_{W^{s,q}({\mathcal {D}})}\\&\quad + {C h^{s'}\Vert f\Vert _{W^{s',2}({\mathcal {D}})}.} \end{aligned}$$

#### Proof

Let us split the error into the following two parts

$$\begin{aligned} u_h^{\text {MC}} - u = u_h^{\text {MC}} - R_h u + R_h u - u =: \theta + \rho , \end{aligned}$$

where $R_h :H^1_0({\mathcal {D}}) \rightarrow S_h$ denotes the Ritz projector (see Section 2). Observe that $\theta$ and $\rho$ are orthogonal with respect to the bilinear form *a*. Then, it follows from the positivity (2.10) and boundedness (2.11) of *a* that

$$\begin{aligned} \sigma _0 |u_h^{\text {MC}} - u|^2_{H^1({\mathcal {D}})}&\le a( u_h^{\text {MC}} - u, u_h^{\text {MC}} - u) = a( \theta , \theta ) + a(\rho , \rho )\\&\le \Vert \sigma \Vert _{L^\infty ({\mathcal {D}})} \big ( |\theta |^2_{H^1({\mathcal {D}})} + |\rho |^2_{H^1({\mathcal {D}})} \big ). \end{aligned}$$

Standard error estimates for the conforming finite element method, cf. (2.16), yield

3.33 $$\begin{aligned} | \rho |_{H^1({\mathcal {D}})} = | R_h u - u|_{H^1({\mathcal {D}})} \le C h \Vert u\Vert _{H^2({\mathcal {D}})}. \end{aligned}$$

Moreover, from (2.12) and (3.24) we get ${\mathbb {P}}$-almost surely for every $v_h \in S_h$ that

$$\begin{aligned} a_{\text {MC}}(\theta , v_h)&= a_{\text {MC}}(u_h^{\text {MC}}, v_h) - a_{\text {MC}}(R_h u, v_h)\\&= F_{\text {MC}}(v_h) - F(v_h) + a(R_h u,v_h) - a_{\text {MC}}(R_h u,v_h), \end{aligned}$$

since $F(v_h) = a(u,v_h) = a(R_h u, v_h)$ for every $v_h \in S_h$. In particular, for the choice $v_h = \theta (\omega ) = u_h^{\text {MC}}(\omega ) - R_h u \in S_h$ we obtain ${\mathbb {P}}$-almost surely that

$$\begin{aligned} \sigma _0 |\theta |^2_{H^1({\mathcal {D}})} \le a_{\text {MC}}(\theta , \theta ) = F_{\text {MC}}(\theta ) - F(\theta ) + a(R_h u, \theta ) - a_{\text {MC}}(R_h u,\theta ). \end{aligned}$$

From Lemma 2 and Theorem 1 it follows directly that all terms on the right-hand side are integrable with respect to ${\mathbb {P}}$. Hence, after taking expectations it remains to give error estimates for the two terms

$$\begin{aligned} E_1&= \big | {\mathbb {E}}\big [ F_{\text {MC}}(\theta ) - F(\theta ) \big ] \big |,\\ E_2&= \big | {\mathbb {E}}\big [ a(R_h u, \theta ) - a_{\text {MC}}(R_h u, \theta ) \big ] \big |. \end{aligned}$$

If $f\in L^{p}({\mathcal {D}})$, $p\in (2,\infty ]$, an application of Lemma 3 directly yields

$$\begin{aligned} E_1 \le {\left\{ \begin{array}{ll} C h^{1 - \frac{2}{p}} \Vert f \Vert _{L^p({\mathcal {D}})} \Vert \theta \Vert _{L^2(\varOmega ;H^1_0({\mathcal {D}}))},& \quad \text {if } p \in [2,\infty ),\\ C \ell _h^{\frac{1}{2}} h \Vert f \Vert _{L^\infty ({\mathcal {D}})} \Vert \theta \Vert _{L^2(\varOmega ;H^1_0({\mathcal {D}}))},& \quad \text {if } p = \infty . \end{array}\right. } \end{aligned}$$

If $f\in W^{s',2}({\mathcal {D}})$, an application of Lemma 4 leads to

$$\begin{aligned} E_1 \le {C h^{s'}\Vert f\Vert _{W^{s',2}({\mathcal {D}})}.} \end{aligned}$$

Next, we turn to the term $E_2$ which is given by

$$\begin{aligned} E_2&= \big | {\mathbb {E}}\big [ a(R_h u,\theta ) - a_{\text {MC}}(R_h u, \theta ) \big ] \big |\\&= \Big | \sum _{T \in \mathcal {T}_h} {\mathbb {E}}\Big [ \int _T \sigma (x) \nabla R_h u(x) \cdot \nabla \theta (x) \,\textrm{d}x - |T| \sigma (Z_T) \nabla R_h u(Z_T) \cdot \nabla \theta (Z_T) \Big ] \Big |. \end{aligned}$$

Since $R_h u \in S_h$ and $\theta :\varOmega \rightarrow S_h$, the respective gradients are constant on each triangle.

Therefore, we have $\nabla R_h u (x) \cdot \nabla \theta (x) = \nabla R_h u (Z_T) \cdot \nabla \theta (Z_T)$ for every $x \in T$. Hence, we get

$$\begin{aligned} E_2&= \Big | \sum _{T \in \mathcal {T}_h} {\mathbb {E}}\Big [ \Big ( \int _T \sigma (x) \,\textrm{d}x - |T| \sigma (Z_T) \Big ) \nabla R_h u(Z_T) \cdot \nabla \theta (Z_T) \Big ] \Big |\\&\le \sum _{T \in \mathcal {T}_h} \Big ( {\mathbb {E}}\Big [ \Big | \Big (\int _T \sigma (x) \,\textrm{d}x - |T| \sigma (Z_T) \Big ) \nabla R_h u(Z_T) \Big |^2 \Big ] \Big )^{\frac{1}{2}} \big ( {\mathbb {E}}\big [ | \nabla \theta (Z_T)|^2 \big ] \big )^{\frac{1}{2}}\\&\le \Big ( \sum _{T \in \mathcal {T}_h} |T|^{-1} {\mathbb {E}}\Big [ \Big ( \int _T \sigma (x) \,\textrm{d}x - |T| \sigma (Z_T) \Big )^2 \big | \nabla R_h u(Z_T)\big |^2 \Big ] \Big )^{\frac{1}{2}}\\&\quad \times \Big ( \sum _{T \in \mathcal {T}_h} |T| {\mathbb {E}}\big [ | \nabla \theta (Z_T) |^2 \big ] \Big )^{\frac{1}{2}} \end{aligned}$$

by further applications of the Cauchy–Schwarz inequality. Moreover, by making again use of the fact that the gradient of $\theta$ is piecewise constant we obtain

$$\begin{aligned} \Big ( \sum _{T \in \mathcal {T}_h} |T| {\mathbb {E}}\big [ | \nabla \theta (Z_T) |^2 \big ] \Big )^{\frac{1}{2}}&= \Big ( {\mathbb {E}}\Big [ \sum _{T \in \mathcal {T}_h} |T| | \nabla \theta (Z_T) |^2 \Big ] \Big )^{\frac{1}{2}}\\&= \Big ( {\mathbb {E}}\Big [ \int _{\mathcal {D}}|\nabla \theta (x)|^2 \,\textrm{d}x \Big ] \Big )^{\frac{1}{2}} = \Vert \theta \Vert _{L^2(\varOmega ;H^1_0({\mathcal {D}}))}. \end{aligned}$$

Further, due to $Z_T \sim |T|^{-1} \mathbb {I}_{T}(z) \,\textrm{d}z$ it holds

3.34 $$\begin{aligned} \begin{aligned}&\Big ( \sum _{T \in \mathcal {T}_h} |T|^{-1} {\mathbb {E}}\Big [ \Big ( \int _T \sigma (x) \,\textrm{d}x - |T| \sigma (Z_T) \Big )^2 | \nabla R_h u(Z_T)|^2 \Big ] \Big )^{\frac{1}{2}}\\&\quad = \Big ( \sum _{T \in \mathcal {T}_h} |T|^{-2} \int _T \Big ( \int _T \big ( \sigma (x) - \sigma (z) \big ) \,\textrm{d}x \Big )^2 | \nabla R_h u(z) |^2 \,\textrm{d}z \Big )^{\frac{1}{2}}\\&\quad \le \Big ( \sum _{T \in \mathcal {T}_h} |T|^{-2} \int _T \Big ( \int _T \big ( \sigma (x) - \sigma (z) \big ) \,\textrm{d}x \Big )^2 | \nabla (R_h - I) u(z) |^2 \,\textrm{d}z \Big )^{\frac{1}{2}}\\&\qquad + \Big ( \sum _{T \in \mathcal {T}_h} |T|^{-2} \int _T \Big ( \int _T \big ( \sigma (x) - \sigma (z) \big ) \,\textrm{d}x \Big )^2 | \nabla u(z) |^2 \,\textrm{d}z \Big )^{\frac{1}{2}}, \end{aligned} \end{aligned}$$

where we applied Minkowski’s inequality in the last step. The first term is then estimated by

$$\begin{aligned}&\Big ( \sum _{T \in \mathcal {T}_h} |T|^{-2} \int _T \Big ( \int _T \big ( \sigma (x) - \sigma (z) \big ) \,\textrm{d}x \Big )^2 | \nabla (R_h - I) u(z) |^2 \,\textrm{d}z \Big )^{\frac{1}{2}}\\&\quad \le \Big ( \sum _{T \in \mathcal {T}_h} |T|^{-1} \int _T \int _T \big ( \sigma (x) - \sigma (z) \big )^2 \,\textrm{d}x | \nabla (R_h - I) u(z) |^2 \,\textrm{d}z \Big )^{\frac{1}{2}}\\&\quad \le C \Vert \sigma \Vert _{L^\infty ({\mathcal {D}})} \Big ( \sum _{T \in \mathcal {T}_h} \int _T | \nabla (R_h - I) u(z) |^2 \,\textrm{d}z \Big )^{\frac{1}{2}}\\&\quad \le C \Vert \sigma \Vert _{L^\infty ({\mathcal {D}})} \big | (R_h - I) u \big |_{H^1({\mathcal {D}})} \le C \Vert \sigma \Vert _{L^\infty ({\mathcal {D}})} \Vert u \Vert _{H^2({\mathcal {D}})} h \end{aligned}$$

by a further application of (3.33).

For the estimate of the last term in (3.34) we first consider $s \in (0,1)$. Applying Hölder’s inequality with exponents $\rho = \frac{q}{2} \in (1,\infty )$ and $\rho ' = \frac{q}{q-2} \in (1,\infty )$ yields

$$\begin{aligned}&\Big ( \sum _{T \in \mathcal {T}_h} |T|^{-2} \int _T \Big ( \int _T \big ( \sigma (x) - \sigma (z) \big ) \,\textrm{d}x \Big )^2 | \nabla u(z) |^2 \,\textrm{d}z \Big )^{\frac{1}{2}}\\&\quad \le \Big ( \sum _{T \in \mathcal {T}_h} |T|^{-2} \Big ( \int _T \Big ( \int _T \big | \sigma (x) - \sigma (z) \big | \,\textrm{d}x \Big )^{2 \rho } \,\textrm{d}z \Big )^{\frac{1}{\rho }} \Big ( \int _T | \nabla u(z) |^{2 \rho '} \,\textrm{d}z \Big )^{\frac{1}{\rho '}} \Big )^{\frac{1}{2}}\\&\quad \le \Big ( \sum _{T \in \mathcal {T}_h} |T|^{- \frac{1}{\rho }} \Big ( \int _T \int _T \big | \sigma (x) - \sigma (z) \big |^{2 \rho } \,\textrm{d}x \,\textrm{d}z \Big )^{\frac{1}{\rho }} \Big ( \int _T | \nabla u(z) |^{2 \rho '} \,\textrm{d}z \Big )^{\frac{1}{\rho '}} \Big )^{\frac{1}{2}}\\&\quad \le \Big ( \sum _{T \in \mathcal {T}_h} |T|^{- 1} \int _T \int _T \big | \sigma (x) - \sigma (z) \big |^{q} \,\textrm{d}x \,\textrm{d}z \Big )^{\frac{1}{q}} \Big ( \sum _{T \in \mathcal {T}_h} \int _T | \nabla u(z) |^{2 \rho '} \,\textrm{d}z \Big )^{\frac{1}{2\rho '}}\\&\quad \le \Big ( \sum _{T \in \mathcal {T}_h} |T|^{-1} h^{2 + qs} \int _T \int _T \frac{\big | \sigma (x) - \sigma (z) \big |^{q}}{|x-z|^{2 +qs}} \,\textrm{d}x \,\textrm{d}z \Big )^{\frac{1}{q}} \Vert u \Vert _{W^{1,2\rho '}({\mathcal {D}})} \end{aligned}$$

since $|x-y| \le h$ for all $x,y \in T$.

Next, recall that the Sobolev embedding theorem [1, Theorem 4.12] yields

$$\begin{aligned} \Vert u \Vert _{W^{1,2\rho '}({\mathcal {D}})} \le C \Vert u \Vert _{H^2({\mathcal {D}})}. \end{aligned}$$

In addition, we have $|T|^{-1} \le c^{-1} h^{-2}$ due to Assumption 2. Altogether, this shows

$$\begin{aligned}&\Big ( \sum _{T \in \mathcal {T}_h} |T|^{-1} h^{2 + qs} \int _T \int _T \frac{\big | \sigma (x) - \sigma (z) \big |^{q}}{|x-z|^{2 +qs}} \,\textrm{d}x \,\textrm{d}z \Big )^{\frac{1}{q}} \Vert u \Vert _{W^{1,2\rho '}({\mathcal {D}})}\\&\quad \le C \Vert u \Vert _{H^2({\mathcal {D}})} | \sigma |_{W^{s,q}({\mathcal {D}})} h^s. \end{aligned}$$

This completes the proof of the case $s \in (0,1)$. The border case $s=1$ follows by similar arguments and an additional application of the Poincaré–Wirtinger inequality. The details are left to the reader. $\square$

## The modified randomized quadrature and its application to Poisson equation

The goal of this section is to increase the accuracy of the randomized quadrature formula $Q_{\text {MC}}$ introduced in (3.19) by applying a standard variance reduction technique for Monte Carlo methods termed *importance sampling*. We briefly review the importance sampling in Section 4.1. Inspired by this technique, we propose a modified randomized quadrature for the load vector (denoted as $F_{IS}$) in Section 4.2, followed by its application to the Poisson equation with a detailed error analysis (see Section 4.3). Note that the randomized quadrature based on importance-sampling for the stiffness matrix coincides with $a_{\text {MC}}$, as discussed in Remark 1. Therefore, considering the analysis of $a_{\text {MC}}$ in Section 3, integrating both $F_{IS}$ and $a_{\text {MC}}$ into the FEM framework for a general elliptic equation is a straightforward extension of the Poisson equation case. To avoid redundancy, we omit this discussion from the paper.

### Importance sampling

An introduction to importance sampling and further variance reduction techniques is found, for instance, in [12, Chapter 6], [29, Chapter 3], and [31, Kapitel 5]. Let us briefly recall the main idea of importance sampling. Suppose one wants to approximate the integral

$$\begin{aligned} \int _{\mathcal {D}}v(x) \,\textrm{d}x, \end{aligned}$$

where $v \in L^2({\mathcal {D}})$ is given. Then, the standard Monte Carlo approach is to rewrite the integral as an expectation

$$\begin{aligned} {\mathbb {E}}[ v(Z) ] = |{\mathcal {D}}|^{-1} \int _{{\mathcal {D}}} v(x) \,\textrm{d}x, \end{aligned}$$

where $Z :\varOmega \rightarrow {\mathcal {D}}$ is a uniformly distributed random variable. In particular, the probability density function of *Z* is given by $p_Z(x) = \frac{1}{|{\mathcal {D}}|}\mathbb {I}_{{\mathcal {D}}}(x)$. Then, the standard Monte Carlo estimator of the integral is defined as

$$\begin{aligned} \frac{|{\mathcal {D}}|}{M} \sum _{i = 1}^{M} v(Z_i), \end{aligned}$$

where $(Z_i)_{i = 1}^{M}$, $M \in {\mathbb {N}}$, is a family of independent and identically distributed copies of *Z*. This estimator is unbiased and its variance is equal to

$$\begin{aligned} \Big \Vert \frac{|{\mathcal {D}}|}{M} \sum _{i = 1}^{M} v(Z_i) - \int _{\mathcal {D}}v(x) \,\textrm{d}x \Big \Vert _{L^2(\varOmega )}^2&= \frac{1}{M} \textrm{var}\big (|{\mathcal {D}}| v(Z)\big ). \end{aligned}$$

Therefore, the accuracy of the Monte Carlo estimator is determined by the number of samples $M \in {\mathbb {N}}$ and the variance of the random variable $|{\mathcal {D}}| v(Z)$.

The main idea of importance sampling is then to increase the accuracy of the standard Monte Carlo estimator by replacing the uniformly distributed random variable *Z* with a random variable $Y :\varOmega \rightarrow {\mathcal {D}}$ whose distribution is determined by a probability distribution function $p_Y$. If the density $p_Y$ satisfies that $p_Y(x) = 0$ only if $v(x) = 0$, then it follows from the transformation theorem that

$$\begin{aligned} \int _{\mathcal {D}}v(x) \,\textrm{d}x = \int _{\mathcal {D}}\frac{v(x)}{p_Y(x)} p_Y(x) \,\textrm{d}x = {\mathbb {E}}_{p_Y} \Big [ \frac{v(Y)}{p_Y(Y)} \Big ], \end{aligned}$$

where we write ${\mathbb {E}}_{p_Y}$ to emphasise the expectation corresponds to distribution $p_Y$.

From this one derives the following *importance sampling estimator* given by

$$\begin{aligned} \frac{1}{M} \sum _{i = 1}^{M} \frac{v(Y_i)}{p_Y(Y_i)}, \end{aligned}$$

where $(Y_i)_{i = 1}^M$ denotes a family of independent and identically distributed copies of *Y*. The art of importance sampling is then to determine a suitable density $p_Y$ such that the variance is reduced and, at the same time, the generation of random variates with density $p_Y$ is computational feasible and affordable. It is known (cf. [12, Theorem 6.5]) that the optimal choice of the density $p_Y$ is

$$\begin{aligned} p_Y^*(x) = \frac{|v(x)|}{\int _{\mathcal {D}}|v(y)|\,\textrm{d}y}, \quad \text { for } x \in {\mathcal {D}}. \end{aligned}$$

Observe that $p_Y^*$ suggests to avoid sampling in regions of $|{\mathcal {D}}|$, where |*v*| is zero or very small. However, since the denominator is typically unknown it is, in general, not possible to use the density $p_Y^*$ in practice.

Nevertheless, one can often still make use of the underlying idea to improve the accuracy of the randomized quadrature rule (3.19).

### The modified randomized quadrature for the load vector

Recall that the entries of the load vector $f_h \in {\mathbb {R}}^{N_h}$ defined in (1.6) are given by

$$\begin{aligned} F(\varphi _j) = \int _{{\mathcal {D}}} f(x) \varphi _j(x) \,\textrm{d}x, \quad j \in \{1,\ldots ,N_h\}, \end{aligned}$$

where $(\varphi _j)_{j = 1}^{N_h}$ denotes the standard Lagrange basis of the finite element space $S_h$. According to the results in the previous section, these entries are then approximated by an application of the randomized quadrature formula (3.19) given by

$$\begin{aligned} F_{\text {{MC}}}( \varphi _j) = Q_{\text {{MC}}}[ f \varphi _j ] = \sum _{T \in \mathcal {T}_h} |T| f(Z_T) \varphi _j(Z_T) \end{aligned}$$

for every $j \in \{1,\ldots ,N_h\}$. Observe that for each triangle $T \in \mathcal {T}_h$ the term

4.35 $$\begin{aligned} |T| f(Z_T) \varphi _j(Z_T) \end{aligned}$$

can be regarded as a standard Monte Carlo estimator with only $M=1$ sample for the integral

$$\begin{aligned} \int _T f(x) \varphi _j(x) \,\textrm{d}x. \end{aligned}$$

The idea of this section is to replace this term by a suitable importance sampling estimator.

Since we do not want to impose any additional assumption on *f* it is, as already mentioned above, not feasible to use the corresponding optimal density function $p_Y^*$ with $v = f \varphi _j$. Instead, we recall that the piecewise linear basis function $\varphi _j$ is equal to zero in two of the three vertices and equal to one in the remaining vertex of every triangle $T \in \mathcal {T}_h$ with $T \cap \textrm{supp}(\varphi _j) \ne \emptyset$. In particular, this implies $\varphi _j(x) \ge 0$ for every $x \in T$. Further, it holds

$$\begin{aligned} \int _T \varphi _j(x) \,\textrm{d}x = \frac{1}{3} |T|. \end{aligned}$$

Therefore, the mapping $p_{T,j} :{\mathcal {D}}\rightarrow [0,\infty )$ defined by

4.36 $$\begin{aligned} p_{T,j}(x) = 3 |T|^{-1} \varphi _j(x) \mathbb {I}_T(x), \quad x \in {\mathcal {D}}, \end{aligned}$$

is a probability density function. By replacing $Z_T$ in (4.35) with a random variable $Y_{T,j} \sim p_{T,j}(x) \,\textrm{d}x$ we arrive at the corresponding importance sampling estimator (again with only $M = 1$ sample)

$$\begin{aligned} \frac{f(Y_{T,j})\varphi _j(Y_{T,j})}{p_{T,j}(Y_{T,j})} = \frac{1}{3} |T| f(Y_{T,j}) \end{aligned}$$

for the integral $\int _T f(x) \varphi _j(x) \,\textrm{d}x$. Observe that the use of $Y_{T,j}$ significantly decreases the probability of the integrand $f \varphi _j$ being evaluated at a point $x \in T$ close to a vertex, where the basis function $\varphi _j$ is equal to zero. We discuss the simulation of the random variable $Y_{T,j}$ in Section 5. Without causing ambiguity, throughout this section we will still adopt ${\mathbb {E}}$ to denote the expectation with respect to $p_{T,j}$.

To sum up, this suggests to use the linear mapping $F_{IS} :S_h \rightarrow L^2(\varOmega )$ given by

4.37 $$\begin{aligned} F_{IS}(v_h) = \frac{1}{3} \sum _{T \in \mathcal {T}_h} |T| \sum _{\begin{array}{c} j = 1\\ T \cap \textrm{supp}(\varphi _j) \ne \emptyset \end{array}}^{N_h} v_j f(Y_{T,j}) \end{aligned}$$

for every $v_h = \sum _{j = 1}^{N_h} v_j \varphi _j \in S_h$. Hereby, $(Y_{T,j})_{T \in \mathcal {T}_h, j \in \{1,\ldots ,N_h\}}$ is a family of independent random variables with $Y_{T,j} \sim p_{T,j}(x) \,\textrm{d}x$. In particular, the entries of the load vector $f_h$ are then approximated by

$$\begin{aligned} F_{IS}(\varphi _j) = \frac{1}{3} \sum _{\begin{array}{c} T \in \mathcal {T}_h\\ T \cap \textrm{supp}(\varphi _j) \ne \emptyset \end{array}} |T| f(Y_{T,j}) \end{aligned}$$

for every $$j \in \{1,\ldots ,N_h\}$$.

#### Remark 1

Note that the entries of the stiffness matrix $A_h$ defined in (1.5) are given by

$$\begin{aligned} a(\varphi _i,\varphi _j) = \int _{\mathcal {D}}\sigma (x) \nabla \varphi _i(x) \cdot \nabla \varphi _j (x) \,\textrm{d}x =\sum _{T\in \mathcal {T}_h } \int _T \sigma (x) \big (\nabla \varphi _i(x) \cdot \nabla \varphi _j (x)\big ) \,\textrm{d}x \end{aligned}$$

for $i,j\in \{1,\ldots ,N_h\}$, where the function $\big (\nabla \varphi _i(\cdot ) \cdot \nabla \varphi _j (\cdot )\big )$ is constant on each *T* and is non-zero constant on each *T* with $T \cap \textrm{supp}(\varphi _i)\cap \textrm{supp}(\varphi _j) \ne \emptyset$. Recall that the (partially) importance sampling strategy described above achieves variance reduction by choosing an appropriate reference density function and assigning higher weights to the region of domain with higher density evaluation. Applying this strategy to approximate the summand of the right hand side through a reference density proportional to $\big (\nabla \varphi _i(\cdot ) \cdot \nabla \varphi _j (\cdot )\big )$ would leads to the estimator $a_{\text {{MC}}}$ as the reference density function being constant over *T*.

Remark 1 demonstrates that the importance sampling strategy does not change the evaluation of stiffness matrix. Since this section focuses on assessing the performance of the modified quadrature (4.37) in comparison to (3.23), we will use the Poisson equation as an example, providing a detailed error analysis.

As the following lemma shows, the importance sampling estimator (4.37) is unbiased and convergent in the limit $h \rightarrow 0$.

#### Lemma 5

Let $\mathcal {T}_h$ be an admissible triangulation with maximal edge length $h \in (0,1]$. Then, for every $f \in L^1({\mathcal {D}})$ and $v_h \in S_h$ it holds that

$$\begin{aligned} {\mathbb {E}}\big [ F_{IS}(v_h) \big ] = \int _{\mathcal {D}}f(x)v_h(x) \,\textrm{d}x. \end{aligned}$$

Further, if $f \in L^p({\mathcal {D}})$, $p \in [2,\infty ]$, then it holds for every $v_h \in S_h$ that

$$\begin{aligned}&\Big \Vert \int _{\mathcal {D}}f(x)v_h(x) \,\textrm{d}x - F_{IS}(v_h) \Big \Vert _{L^2(\varOmega )}\\&\quad \le \frac{1}{\root 4 \of {12}} h \Vert v_h \Vert _{L^\infty ({\mathcal {D}})}^{\frac{2}{p}} \Vert f \Vert _{L^p({\mathcal {D}})} \big ( 2 h |v_h|_{H^1({\mathcal {D}})} + \Vert v_h\Vert _{L^2({\mathcal {D}})} \big )^{1 - \frac{2}{p}}. \end{aligned}$$

In addition, if $f \in W^{s,2}({\mathcal {D}})$ for some $s \in (0,1)$ then it holds for every $v_h \in S_h$ that

$$\begin{aligned} \Big \Vert \int _{\mathcal {D}}f(x)v_h(x) \,\textrm{d}x - F_{IS}(v_h) \Big \Vert _{L^2(\varOmega )} \le h^{1 + s} \Vert v_h\Vert _{L^\infty ({\mathcal {D}})} |f|_{W^{s,2}({\mathcal {D}})}. \end{aligned}$$

#### Proof

Let $v_h = \sum _{j = 1}^{N_h} v_j \varphi _j \in S_h$ be arbitrary with coefficients $(v_j)_{j = 1}^{N_h} \subset {\mathbb {R}}$. Due to $Y_{T,j} \sim \frac{3}{|T|}\varphi _j(z) \mathbb {I}_T(z) \,\textrm{d}z$ for every $T \in \mathcal {T}_h$ we have

$$\begin{aligned} \sum _{j = 1}^{N_h} v_j {\mathbb {E}}\Big [ \frac{ |T|}{3} f(Y_{T,j}) \Big ] = \sum _{j = 1}^{N_h} v_j \frac{|T|}{3} \int _T f(z)\varphi _j(z) \frac{3}{|T|} \,\textrm{d}z = \int _T f(z) v_h(z) \,\textrm{d}z. \end{aligned}$$

Then, the first assertion follows by summing over all triangles of the triangulation.

Now, let $f \in L^2({\mathcal {D}})$ be arbitrary. In the same way as in the proof of Lemma 1, the mean-square error is shown to be equal to

$$\begin{aligned}&\Big \Vert \int _{\mathcal {D}}f(x) v_h(x) \,\textrm{d}x - F_{IS}(v_h) \Big \Vert _{L^2(\varOmega )}^2\\&\quad = \sum _{T \in \mathcal {T}_h} \sum _{\begin{array}{c} j = 1\\ T \cap \textrm{supp}(\varphi _j) \ne \emptyset \end{array}}^{N_h} v_j^2{\mathbb {E}}\Big [ \Big | \int _T f(x)\varphi _j(x) \,\textrm{d}x - \frac{|T|}{3} f(Y_{T,j}) \Big |^2 \Big ], \end{aligned}$$

due to the independence of the random variables $(Y_{T,j})_{T \in \mathcal {T}_h, j \in \{1,\ldots ,N_h\}}$.

Then, for every $j\in \{1,\ldots ,N_h\}$ and $T \in \mathcal {T}_h$ with $T \cap \textrm{supp}(\varphi _j)\ne \emptyset$ we make use of $Y_{T,j} \sim \frac{3}{|T|} \mathbb {I}_T(z)\varphi _j(z) \,\textrm{d}z$ and the Cauchy–Schwarz inequality. This yields

$$\begin{aligned}&{\mathbb {E}}\Big [ \Big | \int _T f(x)\varphi _j(x) \,\textrm{d}x - \frac{|T|}{3} f(Y_{T,j}) \Big |^2 \Big ]\\&\quad = \frac{3}{|T|}\int _T \Big |\int _T f(x)\varphi _j(x) \,\textrm{d}x - \frac{|T|}{3} f(z) \Big |^2 \varphi _j(z) \,\textrm{d}z \\&\quad = \frac{3}{|T|}\int _T \Big |\int _T (f(x)-f(z))\varphi _j(x) \,\textrm{d}x \Big |^2\varphi _j(z) \,\textrm{d}z \\&\quad \le \int _T\int _T (f(x)-f(z))^2\varphi _j(x)\varphi _j(z) \,\textrm{d}x\,\textrm{d}z\\&\quad = \frac{2}{3} |T| \int _T |f(x)|^2 \varphi _j(x) \,\textrm{d}x - 2 \Big ( \int _T f(x) \varphi _j(x) \,\textrm{d}x \Big )^2. \end{aligned}$$

We neglect the last term and insert this estimate into the mean-square error. An application of Weitzenböck’s inequality (3.21) then yields

4.38 $$\begin{aligned} \begin{aligned}&\Big \Vert \int _{\mathcal {D}}f(x)v_h(x) \,\textrm{d}x - F_{IS}(v_h) \Big \Vert _{L^2(\varOmega )}^2\\&\quad = \sum _{T \in \mathcal {T}_h} \sum _{\begin{array}{c} j = 1\\ T \cap \textrm{supp}(\varphi _j) \ne \emptyset \end{array}}^{N_h} v_j^2 {\mathbb {E}}\Big [ \Big | \int _T f(x)\varphi _j(x) \,\textrm{d}x - \frac{|T|}{3} f(Y_{T,j}) \Big |^2 \Big ]\\&\quad \le \frac{2}{3} \sum _{T \in \mathcal {T}_h} \sum _{\begin{array}{c} j = 1\\ T \cap \textrm{supp}(\varphi _j) \ne \emptyset \end{array}}^{N_h} v_j^2 |T| \int _T | f(x) |^2 \varphi _j(x) \,\textrm{d}x\\&\quad \le \frac{1}{2\sqrt{3}} h^2 \sum _{T \in \mathcal {T}_h} \sum _{\begin{array}{c} j = 1\\ T \cap \textrm{supp}(\varphi _j) \ne \emptyset \end{array}}^{N_h} v_j^2 \int _T | f(x) |^2 \varphi _j(x) \,\textrm{d}x. \end{aligned} \end{aligned}$$

Now, we assume that $f \in L^p({\mathcal {D}})$ with $p \in [2,\infty ]$. To every $v_h = \sum _{j = 1}^{N_h} v_j \varphi _j \in S_h$ we then associate a mapping ${v}^\circ _h :{\mathcal {D}}\rightarrow {\mathbb {R}}$ defined by $v^\circ _h(x) = \sum _{T \in \mathcal {T}_h} v_T \mathbb {I}_T(x)$, where $v_T := v_h(z_T)$ and $z_T \in T$ denotes the barycenter of $T \in \mathcal {T}_h$. Observe that $v^\circ _h$ is piecewise constant on each triangle.

For every $T \in \mathcal {T}_h$ and $j \in \{1,\ldots ,N_h\}$ with $T \cap \textrm{supp}(\varphi _j) \ne \emptyset$ let $z_j \in \overline{T}$ be the uniquely determined node, which satisfies $\varphi _j(z_j)=1$. Clearly, it holds $|z_j - z_T| \le h$. Since $v_h$ is affine linear we obtain that

$$\begin{aligned} |v_j - v_T| = |v_h(z_j) - v_h(z_T)| \le | \nabla v_h(z_T)| h. \end{aligned}$$

Then, we continue the estimate of the mean-square error in (4.38) by adding and subtracting the coefficients of $v_h^\circ$ as follows: For $\rho = \frac{p}{2} \in [1,\infty ]$ let $\rho ' = \frac{p}{p-2} \in [1,\infty ]$ be the conjugated Hölder exponent determined by $\frac{1}{\rho } + \frac{1}{\rho '} = 1$, where we set $\frac{1}{\infty } = 0$. Then, we get

$$\begin{aligned} v_j^2&= |v_j|^{\frac{2}{\rho }} |v_j|^{\frac{2}{\rho '}} \le \max _i |v_i|^{ \frac{2}{\rho }} \big ( |v_j - v_T| + |v_T| \big )^{\frac{2}{\rho '}}\\&\le \Vert v_h \Vert _{L^\infty ({\mathcal {D}})}^{\frac{2}{\rho }} \big ( |\nabla v_h(z_T)| h + |v_T| \big )^{\frac{2}{\rho '}}. \end{aligned}$$

After inserting this into (4.38) we obtain

$$\begin{aligned}&\Big \Vert \int _{\mathcal {D}}f(x) v_h(x) \,\textrm{d}x - F_{IS}(v_h) \Big \Vert _{L^2(\varOmega )}^2\\&\; \le \frac{1}{2\sqrt{3}} h^2 \Vert v_h \Vert _{L^\infty ({\mathcal {D}})}^{\frac{2}{\rho }} \sum _{T \in \mathcal {T}_h} \sum _{\begin{array}{c} j = 1\\ T \cap \textrm{supp}(\varphi _j) \ne \emptyset \end{array}}^{N_h} \big ( |\nabla v_h(z_T)| h + |v_T| \big )^{\frac{2}{\rho '}} \int _T | f(x) |^2 \varphi _j(x) \,\textrm{d}x\\&\; \le \frac{1}{2\sqrt{3}} h^2 \Vert v_h \Vert _{L^\infty ({\mathcal {D}})}^{\frac{2}{\rho }} \int _{\mathcal {D}}\big ( |\nabla v_h(x)| h + |v_h^\circ (x)| \big )^{\frac{2}{\rho '}} | f(x) |^2 \,\textrm{d}x, \end{aligned}$$

since $\nabla v_h$ and $v_h^\circ$ are constant on each *T*. In addition, we also made use of

4.39 $$\begin{aligned} 0 \le \sum _{j = 1}^{N_h} \varphi _{j}(x) \le 1 \end{aligned}$$

for every $$x \in \overline{{\mathcal {D}}}$$.

Therefore, applications of Hölder’s inequality and Minkowski’s inequality yield

4.40 $$\begin{aligned} \begin{aligned}&\Big \Vert \int _{\mathcal {D}}f(x) v_h(x) \,\textrm{d}x - F_{IS}(v_h) \Big \Vert _{L^2(\varOmega )}^2\\&\quad \le \frac{1}{2\sqrt{3}} h^2 \Vert v_h \Vert _{L^\infty ({\mathcal {D}})}^{\frac{2}{\rho }} \Vert f \Vert _{L^p({\mathcal {D}})}^{2} \Big ( \int _{\mathcal {D}}\big ( |\nabla v_h(x)| h + |v_h^\circ (x)| \big )^{2} \,\textrm{d}x \Big )^{\frac{1}{\rho '}}\\&\quad \le \frac{1}{2\sqrt{3}} h^2 \Vert v_h \Vert _{L^\infty ({\mathcal {D}})}^{\frac{2}{\rho }} \Vert f \Vert _{L^p({\mathcal {D}})}^{2} \big ( h |v_h|_{H^1({\mathcal {D}})} + \Vert v_h^\circ \Vert _{L^2({\mathcal {D}})} \big )^{\frac{2}{\rho '}}. \end{aligned} \end{aligned}$$

Finally, we observe that

$$\begin{aligned} \Vert v_h - v_h^\circ \Vert _{L^2({\mathcal {D}})}^2&= \sum _{T \in \mathcal {T}_h} \int _T | v_h(x) - v_T|^2 \,\textrm{d}x\\&= \sum _{T \in \mathcal {T}_h} \int _T | \nabla v_h(x) \cdot (x - z_T) |^2 \,\textrm{d}x \le h^2 | v_h |_{H^1({\mathcal {D}})}^2 \end{aligned}$$

since $|x - z_T|\le h$ for every $x \in T$ and $\nabla v_h$ is piecewise constant on *T*. Consequently,

$$\begin{aligned} \Vert v_h^\circ \Vert _{L^2({\mathcal {D}})} \le \Vert v_h^\circ - v_h\Vert _{L^2({\mathcal {D}})} + \Vert v_h\Vert _{L^2({\mathcal {D}})} \le h | v_h |_{H^1({\mathcal {D}})} + \Vert v_h\Vert _{L^2({\mathcal {D}})}. \end{aligned}$$

Inserting this into (4.40) then completes the proof of the second assertion.

To prove the third assertion let $f \in W^{s,2}({\mathcal {D}})$, $s\in (0,1)$. As above we have

$$\begin{aligned}&\Big \Vert \int _{\mathcal {D}}f(x) v_h(x) \,\textrm{d}x - F_{IS}(v_h) \Big \Vert _{L^2(\varOmega )}^2\\&\quad \le \sum _{T \in \mathcal {T}_h} \sum _{\begin{array}{c} j = 1\\ T \cap \textrm{supp}(\varphi _j) \ne \emptyset \end{array}}^{N_h} v_j^2 \int _T\int _T (f(x)-f(z))^2\varphi _j(x)\varphi _j(z) \,\textrm{d}x\,\textrm{d}z\\&\quad \le \max _{i} |v_i|^2 \sum _{T \in \mathcal {T}_h} \int _T \int _T (f(x)-f(z))^2 \,\textrm{d}x\,\textrm{d}z, \end{aligned}$$

where we also used that $\varphi _j(z) \le 1$ for all $z \in T$ and (4.39). Moreover, since $f \in W^{s,2}({\mathcal {D}})$ we get

$$\begin{aligned} \sum _{T \in \mathcal {T}_h} \int _T \int _T (f(x)-f(z))^2 \,\textrm{d}x\,\textrm{d}z&\le h^{2(1 + s)} \sum _{T \in \mathcal {T}_h} \int _T \int _T \frac{ |f(x)-f(z)|^2}{|x-z|^{2 + 2s}} \,\textrm{d}x\,\textrm{d}z\\&\le h^{2(1 + s)} |f|_{W^{s,2}({\mathcal {D}})}^2. \end{aligned}$$

Altogether, this completes the proof of the third assertion. $\square$

The well-posedness of (4.37) is a consequence of Lemma 5. The following lemma contains some further estimates of $F_{IS}$ provided the family of triangulations satisfies Assumption 2.

#### Corollary 1

Suppose that $f \in L^2({\mathcal {D}})$. Let $(\mathcal {T}_{h})_{h \in (0,1]}$ be a family of triangulations satisfying Assumption 2. Then, there exists $C \in (0,\infty )$ independent of $\mathcal {T}_h$ such that

$$\begin{aligned} |F_{IS}(v_h)|&\le C \ell _h^{\frac{1}{2}} \bar{F}_{IS,h} |v_h|_{H^1({\mathcal {D}})} < \infty \quad {\mathbb {P}}\text { -a.s.},\\ \Vert F_{IS}(v_h) \Vert _{L^2(\varOmega )}&\le C \Vert f \Vert _{L^2({\mathcal {D}})} |v_h|_{H^1({\mathcal {D}})} , \end{aligned}$$

for all $v_h \in S_h$, where $\ell _h = \max ( 1, \log (1/h))$ and $\bar{F}_{IS,h} :\varOmega \rightarrow {\mathbb {R}}$ is defined as

$$\begin{aligned} \bar{F}_{IS,h} := \frac{1}{3} \sum _{T \in \mathcal {T}_h} |T| \sum _{\begin{array}{c} j = 1 \\ T \cap \textrm{supp}(\varphi _j) \ne \emptyset \end{array}}^{N_h} |f(Y_{T,j})|. \end{aligned}$$

#### Proof

We only verify the almost sure bound for $F_{IS}(v_h)$. The estimate of the $L^2(\varOmega )$-norm then follows from Lemma 5 and the same arguments as in the proof Lemma 2.

By the definition of $F_{IS}$ and an application of (2.13) we have that

$$\begin{aligned} | F_{IS}(v_h) |&\le \frac{1}{3} \sum _{T \in \mathcal {T}_h} |T| \sum _{\begin{array}{c} j = 1\\ T \cap \textrm{supp}(\varphi _j) \ne \emptyset \end{array}}^{N_h} |v_j| |f(Y_{T,j})|\\&\le \frac{1}{3} \Vert v_h \Vert _{L^\infty ({\mathcal {D}})} \sum _{T \in \mathcal {T}_h} |T| \sum _{\begin{array}{c} j = 1\\ T \cap \textrm{supp}(\varphi _j) \ne \emptyset \end{array}}^{N_h} |f(Y_{T,j})|\\&\le C \ell _h^{\frac{1}{2}} | v_h |_{H^1({\mathcal {D}})} \bar{F}_{IS,h}. \end{aligned}$$

It remains to show that $\bar{F}_{IS,h}$ is bounded $\mathbb {P}$-almost surely. But this follows immediately from

$$\begin{aligned} {\mathbb {E}}\big [ \bar{F}_{IS,h} \big ]&= \frac{1}{3} \sum _{T \in \mathcal {T}_h} |T| \sum _{\begin{array}{c} j = 1 \\ T \cap \textrm{supp}(\varphi _j) \ne \emptyset \end{array}}^{N_h} {\mathbb {E}}\big [ |f(Y_{T,j})| \big ]\\&= \sum _{T \in \mathcal {T}_h} \sum _{\begin{array}{c} j = 1 \\ T \cap \textrm{supp}(\varphi _j) \ne \emptyset \end{array}}^{N_h} \int _T |f(y)| \varphi _j(y) \,\textrm{d}y\\&\le \int _{\mathcal {D}}|f(y)| \,\textrm{d}y < \infty , \end{aligned}$$

where we used that $\sum _{j = 1}^{N_h} \varphi _j(y) \le 1$ for every $y \in {\mathcal {D}}$. In turn, this implies $\bar{F}_{IS,h}< \infty$ $\mathbb {P}$-almost surely. $\square$

### Its application to the Poisson equation

To demonstrate the performance of the modified quadrature in (4.37), we solely focus on the Poisson equation

4.41 $$\begin{aligned} {\left\{ \begin{array}{ll} - \varDelta u = f,&  \quad \text { in } {\mathcal {D}},\\ u = 0,&  \quad \text { on } \partial {\mathcal {D}}, \end{array}\right. } \end{aligned}$$

where ${\mathcal {D}}\subset {\mathbb {R}}^2$ is a convex, bounded and polygonal domain and $f \in L^p({\mathcal {D}})$ for some $p\in [2,\infty ]$.

Observe that the Poisson equation is a particular case of the boundary value problem (1.1) with $\sigma \equiv 1$. In this case, the assembly of the stiffness matrix $A_h$ in (1.4) does not require the application of a (randomized) quadrature rule. Therefore the term $a(\cdot ,\cdot )$ remains when introducing the corresponding FEM in (4.42).

Next, we introduce the finite element problem based on the importance sampling estimator. In terms of $F_{IS}$ the problem is stated as follows:

4.42 $$\begin{aligned} {\left\{ \begin{array}{ll} \text {Find } u_{h}^{IS} :\varOmega \rightarrow S_{h} \text { such that } {\mathbb {P}}\text {-almost surely} \\ a(u_{h}^{IS},v_{h}) = F_{IS}(v_h) \text { for all } v_{h} \in S_h. \end{array}\right. } \end{aligned}$$

In the same way as in Theorem 1 one shows that the discrete problem (4.42) to Poisson equation (4.41) has a uniquely determined solution $u_h^{IS} :\varOmega \rightarrow S_h$.

#### Theorem 3

For every admissible triangulation $\mathcal {T}_h$, $h \in (0,1]$, there exists a uniquely determined measurable mapping $u_h^{IS} :\varOmega \rightarrow S_h$ which solves the discrete problem (4.42). In addition, there exists $C \in (0,\infty )$ independent of $\mathcal {T}_h$ such that

$$\begin{aligned} \big |u_h^{IS}\big |_{H^1({\mathcal {D}})}&\le C \ell _h^{\frac{1}{2}} \bar{F}_{IS,h} \quad {\mathbb {P}}\text {-a.s.,} \end{aligned}$$

where $$\ell _h = \max (1,\log (1/h))$$.

The following theorem contains an estimate of the total error of the approximation $u_h^{IS}$ with respect to the $L^2(\varOmega ;H^1_0({\mathcal {D}}))$-norm.

#### Theorem 4

Let Assumptions 1 and 2 be satisfied. If $f \in L^p({\mathcal {D}})$, $p \in (2,\infty ]$, then there exists $C \in (0,\infty )$ such that for every $h \in (0,1]$

$$\begin{aligned} \big \Vert u_h^{IS} - u \big \Vert _{L^2(\varOmega ;H^1_0({\mathcal {D}}))}&\le C h \Vert u \Vert _{H^2({\mathcal {D}})} + C \ell _h^{\frac{1}{2} + \frac{1}{p}} h^{1 - \frac{2}{p}} \Vert f \Vert _{L^p({\mathcal {D}})}, \end{aligned}$$

where $$\ell _h = \max ( 1, \log (1/h))$$.

#### Proof

As in the proof of Theorem 2 we split the error into the two parts

$$\begin{aligned} u_h^{IS} - u = u_h^{IS} - R_h u + R_h u - u =: \theta + \rho , \end{aligned}$$

where we recall the definition of the Ritz projector $R_h :H^1_0({\mathcal {D}}) \rightarrow S_h$ from Section 2. Since the associated bilinear form *a* for (4.41) coincides with the inner product in $H^1_0({\mathcal {D}})$ it follows that

$$\begin{aligned} \big |u_h^{IS} - u \big |^2_{H^1({\mathcal {D}})}&= a( u_h^{IS} - u, u_h^{IS} - u) = a( \theta , \theta ) + a(\rho , \rho )\\&= |\theta |^2_{H^1({\mathcal {D}})} + |\rho |^2_{H^1({\mathcal {D}})}. \end{aligned}$$

Then, due to (2.16) it holds

$$\begin{aligned} | \rho |_{H^1({\mathcal {D}})} = | R_h u - u|_{H^1({\mathcal {D}})} \le C h \Vert u\Vert _{H^2({\mathcal {D}})}. \end{aligned}$$

Further, from the variational formulation of (4.41) and (4.42) we get ${\mathbb {P}}$-almost surely for every $v_h \in S_h$ that

$$\begin{aligned} a(\theta , v_h)&= a(u_h^{IS}, v_h) - a(R_h u, v_h)\\&= F_{IS}(v_h) - F(v_h), \end{aligned}$$

since $a(R_h u, v_h)= a(u,v_h) = F(v_h)$ for every $v_h \in S_h$. In particular, for the choice $v_h = \theta (\omega ) = u_h^{IS}(\omega ) - R_h u \in S_h$ we obtain ${\mathbb {P}}$-almost surely that

$$\begin{aligned} |\theta |^2_{H^1({\mathcal {D}})} = a(\theta , \theta ) = F_{IS}(\theta ) - F(\theta ). \end{aligned}$$

From Corollary 1 and Theorem 3 it follows directly that all terms on the right-hand side are integrable with respect to ${\mathbb {P}}$. Hence, after taking expectations it remains to prove an estimate for the term

$$\begin{aligned} E_{IS}&= \big | {\mathbb {E}}\big [ F_{IS}(\theta ) - F(\theta ) \big ] \big |. \end{aligned}$$

This is accomplished by the same arguments as in the proof of Lemma 3. More precisely, we represent $\theta$ in terms of an orthonormal basis $(\psi _j)_{j = 1}^{N_h} \subset S_h$ by

$$\begin{aligned} \theta = \sum _{j = 1}^{N_h} \theta _j \psi _j, \end{aligned}$$

where $\theta _j= (\theta , \psi _j)_{L^2({\mathcal {D}})}$, $j = 1, \ldots , N_h$, are real-valued and square-integrable random variables. Hereby, we assume again that $(\psi _j)_{j = 1}^{N_h}$ is a solution to the discrete eigenvalue problem (3.26). Then, by the linearity of *F* and $F_{IS}$ and the Cauchy–Schwarz inequality we obtain the estimate

$$\begin{aligned} E_{IS}&= \Big | {\mathbb {E}}\Big [ \sum _{j = 1}^{N_h} \theta _j \big ( F_{IS}(\psi _j) - F(\psi _j) \big ) \Big ] \Big |\\&\le \Big (\sum _{j =1}^{N_h} \lambda _{h,j} {\mathbb {E}}\big [ |\theta _j|^2 \big ] \Big )^{\frac{1}{2}} \Big ( \sum _{j =1}^{N_h} \lambda _{h,j}^{-1} {\mathbb {E}}\big [ \big | F_{IS}(\psi _j) - F(\psi _j) \big |^2 \big ] \Big )^{\frac{1}{2}}, \end{aligned}$$

where $(\lambda _{h,j})_{j = 1}^{N_h} \subset (0,\infty )$ denote the discrete eigenvalues in (3.26). Then, as in (3.28) one computes

$$\begin{aligned} \Big (\sum _{j =1}^{N_h} \lambda _{h,j} {\mathbb {E}}\big [ |\theta _j|^2 \big ] \Big )^{\frac{1}{2}} = \Vert \theta \Vert _{L^2(\varOmega ;H^1_0({\mathcal {D}}))}. \end{aligned}$$

Moreover, since $f \in L^p({\mathcal {D}})$ and $\Vert \psi _j\Vert _{L^2({\mathcal {D}})}=1$ it follows from Lemma 5 that

$$\begin{aligned} {\mathbb {E}}\big [ \big | F_{IS}(\psi _j) - F(\psi _j) \big |^2 \big ] \le \frac{1}{\sqrt{12}} h^2 \Vert f\Vert _{L^p({\mathcal {D}})}^{2} \Vert \psi _j \Vert ^{\frac{4}{p}}_{L^\infty ({\mathcal {D}})} \big ( 2 h |\psi _j|_{H^1({\mathcal {D}})} + 1 \big )^{2 - \frac{4}{p}}. \end{aligned}$$

Next, we recall from (2.13) and (2.14) that $\Vert \psi _j \Vert _{L^\infty ({\mathcal {D}})} \le C \ell _h^{\frac{1}{2}} |\psi _j|_{H^1({\mathcal {D}})} \le C \ell _h^{\frac{1}{2}} h^{-1}$ for every $j \in \{1,\ldots ,N_h\}$, since $\Vert \psi _j\Vert _{L^2({\mathcal {D}})} = 1$. Therefore,

$$\begin{aligned} {\mathbb {E}}\big [ \big | F_{IS}(\psi _j) - F(\psi _j) \big |^2 \big ] \le C \ell _h^{\frac{2}{p}} h^{2 - \frac{4}{p}} \Vert f\Vert _{L^p({\mathcal {D}})}^{2} \end{aligned}$$

for some constant $C \in (0,\infty )$ independent of $h \in (0,1]$ and $j \in \{1,\ldots ,N_h\}$.

Altogether, we have shown that

$$\begin{aligned} E_{IS} \le C \ell _h^{\frac{1}{p}} h^{1 - \frac{2}{p}} \Vert f\Vert _{L^p({\mathcal {D}})} \Vert \theta \Vert _{L^2(\varOmega ;H^1_0({\mathcal {D}}))} \Big ( \sum _{j = 1}^{N_h} \lambda _{h,j}^{-1} \Big )^{\frac{1}{2}}. \end{aligned}$$

Together with (3.30) this completes the proof. $\square$

Finally, we show an error estimate with respect to the norm in $L^2(\varOmega ;L^2({\mathcal {D}}))$.

#### Theorem 5

Let Assumptions 1 and 2 be satisfied. If $f \in W^{s,2}({\mathcal {D}})$, $s \in [0,1)$, then there exists $C \in (0,\infty )$ such that for every $h \in (0,1]$

$$\begin{aligned} \big \Vert u_h^{IS} - u \big \Vert _{L^2(\varOmega ;L^2({\mathcal {D}}))}&\le C h^2 \Vert u \Vert _{H^2({\mathcal {D}})} + C \ell _h h^{1 + s} | f |_{W^{s,2}({\mathcal {D}})}, \end{aligned}$$

where $$\ell _h = \max ( 1, \log (1/h))$$.

#### Proof

As in the proof of Theorem 4 we again split the error into the two parts

$$\begin{aligned} u_h^{IS} - u = u_h^{IS} - R_h u + R_h u - u =: \theta + \rho . \end{aligned}$$

Then, it follows from (2.17) that

$$\begin{aligned} \Vert \rho \Vert _{L^2({\mathcal {D}})} = \Vert (R_h - I) u \Vert _{L^2({\mathcal {D}})} \le C h^2 \Vert u \Vert _{H^2({\mathcal {D}})} \end{aligned}$$

for every $$h \in (0,1]$$.

In order to give an estimate of the $L^2(\varOmega ;L^2({\mathcal {D}}))$-norm of $\theta$ we apply Nitsche’s duality trick. More precisely, we consider the auxiliary problem of finding a random mapping $w_h :\varOmega \rightarrow S_h$ satisfying ${\mathbb {P}}$-almost surely

4.43 $$\begin{aligned} a(v_h,w_h) = (\theta , v_h)_{L^2({\mathcal {D}})}, \quad \text { for all } v_h \in S_h. \end{aligned}$$

Observe that (4.43) is a linear variational problem with a random right-hand side. The existence of a uniquely determined solution $w_h :\varOmega \rightarrow S_h$ can be shown in the same way as in the proof of Theorem 1.

Testing (4.43) with $v_h = \theta (\omega ) \in S_h$ then gives for ${\mathbb {P}}$-almost every $\omega \in \varOmega$ that

$$\begin{aligned} \Vert \theta (\omega ) \Vert _{L^2({\mathcal {D}})}^2&= a(\theta (\omega ),w_h(\omega )) = a(u_h^{IS}(\omega ), w_h(\omega )) - a(R_h u, w_h(\omega ))\\&= F_{IS}(w_h(\omega )) - F(w_h(\omega )), \end{aligned}$$

where we also applied (4.42), (2.12), and (2.15). Therefore, we have

$$\begin{aligned} \Vert \theta \Vert _{L^2(\varOmega ;L^2({\mathcal {D}}))}^2 = \big | {\mathbb {E}}\big [ F_{IS}(w_h) - F(w_h) \big ] \big |. \end{aligned}$$

Then, as in the proof of Lemma 3 we represent $w_h$ in terms of the orthonormal basis $(\psi _j)_{j = 1}^{N_h}$ consisting of discrete eigenfunctions to the eigenvalue problem (3.26). After inserting this into the $L^2(\varOmega ;L^2({\mathcal {D}}))$-norm of $\theta$, an application of the Cauchy–Schwarz inequality yields

$$\begin{aligned} \Vert \theta \Vert _{L^2(\varOmega ;L^2({\mathcal {D}}))}^2&= \Big | {\mathbb {E}}\Big [ \sum _{j = 1}^{N_h} w_j \big ( F_{IS}( \psi _j) - F(\psi _j) \big ) \Big ] \Big |\\&\le \Big (\sum _{j =1}^{N_h} \lambda _{h,j}^2 {\mathbb {E}}\big [ |w_j|^2 \big ] \Big )^{\frac{1}{2}} \Big ( \sum _{j =1}^{N_h} \lambda _{h,j}^{-2} {\mathbb {E}}\big [ \big | F_{IS}(\psi _j) - F(\psi _j) \big |^2 \big ] \Big )^{\frac{1}{2}}, \end{aligned}$$

where $w_j = (\psi _j,w_h)_{L^2({\mathcal {D}})}$, $j \in \{1,\ldots ,N_h\}$, and $(\lambda _{h,j})_{j = 1}^{N_h} \subset (0,\infty )$ are the discrete eigenvalues in (3.26).

Then, it follows from (3.26), (4.43) and Parseval’s identity that

$$\begin{aligned} \Big (\sum _{j =1}^{N_h} \lambda _{h,j}^2 {\mathbb {E}}\big [ |w_j|^2 \big ] \Big )^{\frac{1}{2}}&= \Big (\sum _{j =1}^{N_h} {\mathbb {E}}\big [ \big | \lambda _{h,j} (\psi _j, w_h)_{L^2({\mathcal {D}})} \big |^2 \big ] \Big )^{\frac{1}{2}}\\&= \Big (\sum _{j =1}^{N_h} {\mathbb {E}}\big [ \big | a(\psi _j, w_h) \big |^2 \big ] \Big )^{\frac{1}{2}}\\&= \Big (\sum _{j =1}^{N_h} {\mathbb {E}}\big [ \big | (\theta ,\psi _j)_{L^2({\mathcal {D}})} \big |^2 \big ] \Big )^{\frac{1}{2}} = \Vert \theta \Vert _{L^2(\varOmega ;L^2({\mathcal {D}}))}. \end{aligned}$$

Hence, this term can be cancelled from both sides of the inequality.

Furthermore, an application of Lemma 5 shows that

4.44 $$\begin{aligned} {\mathbb {E}}\big [ \big | F_{IS}(\psi _j) - F(\psi _j) \big |^2 \big ]&\le h^{2(1+s)} \Vert \psi _j \Vert _{L^\infty ({\mathcal {D}})}^2 | f |_{W^{s,2}({\mathcal {D}})}^2. \end{aligned}$$

After recalling from (2.13) and (3.26) that

$$\begin{aligned} \Vert \psi _j \Vert _{L^\infty ({\mathcal {D}})}^2 \le C \ell _h | \psi _j|_{H^1({\mathcal {D}})}^2 = C \ell _h a(\psi _j,\psi _j) = C \ell _h \lambda _{h,j} \end{aligned}$$

for every $j\in \{1,\ldots ,N_h\}$, we finally arrive at

$$\begin{aligned} \Big ( \sum _{j =1}^{N_h} \lambda _{h,j}^{-2} {\mathbb {E}}\big [ \big | F_{IS}(\psi _j) - F(\psi _j) \big |^2 \big ] \Big )^{\frac{1}{2}}&\le C \ell _h^{\frac{1}{2}} h^{1+s} | f |_{W^{s,2}({\mathcal {D}})} \Big ( \sum _{j = 1}^{N_h} \lambda _{h,j}^{-1} \Big )^{\frac{1}{2}}\\&\le C \ell _h h^{1+s} | f |_{W^{s,2}({\mathcal {D}})}, \end{aligned}$$

where we also inserted (3.30) in the last step. Altogether, this completes the proof for $s\in (0,1)$. The border case $s =0$ is proven analogously. $\square$

#### Remark 2

Note that even if one applies $F_{MC}$ defined in (3.23) instead of $F_{IS}$, the same rate of convergence in Theorem 5 can be obtained for solving the Poisson equation (4.41), where one may need to use Lemma 1 in (4.44). When $s=0$, i.e., $f\in L^2({\mathcal {D}})$, the coefficient of the approximation error bound of $u_h^{IS}$ is smaller than the Monte-Carlo counterpart.

## Implementation of the randomized quadrature formulas

This section is devoted to a brief instruction on how to implement the randomized quadrature formulas (3.19) and (4.37).

To be more precise, we apply the *general rejection algorithm* to sample the random variables $Y_{T,j} \sim p_{T,j}(x) \,\textrm{d}x$ introduced in (4.36) for each element $T \in \mathcal {T}_h$ and $j \in \{1,\ldots ,N_h\}$. We briefly review the rejection algorithm in Section 5.1. To simplify its implementation it is convenient to use a change of coordinates such that the sampling can be done on a fixed reference triangle. This will be discussed in detail in Section 5.2. In Section 5.3 we then show how the required samples are generated on the reference triangle using the rejection algorithm. Moreover, Section 5.4 briefly considers the uniform sampling of $Z_T \sim \mathcal {U}(T)$ on an arbitrary triangle $T \in \mathcal {T}_h$. Finally, in Section 5.5 we sketch how the randomized quadrature formula (3.19) can be embedded into the finite element method.

### General rejection algorithm

In this subsection we briefly recall the general rejection algorithm for the simulation of a non-uniformly distributed random variable whose distribution is given by a probability density function. For more details on this method we refer to [29, Chapter 2.3.2].

For $d \in {\mathbb {N}}$ let $p :\mathbb {R}^d \rightarrow \mathbb {R}$ be a given probability density function. The goal is to generate samples of a random variable $X :\varOmega \rightarrow {\mathbb {R}}^d$ whose distribution is given by $p(x) \,\textrm{d}x$. To this end, we assume that we already know how to generate samples of a random variable $Z :\varOmega \rightarrow {\mathbb {R}}^d$ which is distributed according to a further probability density function $g :{\mathbb {R}}^d \rightarrow {\mathbb {R}}$. Suppose that there exists $c \in (0,\infty )$ such that

5.45 $$\begin{aligned} p(x) \le c g(x),\quad \text { for all } x\in \mathbb {R}^d. \end{aligned}$$

Then, the *general rejection algorithm* is given by:

1. Generate a sample $$Z \sim g(x)\,\textrm{d}x$$.
2. Generate a sample $Y\sim \mathcal {U}(0,c)$ independently from *Z*.
3. Return the value of *Z* if $Y\cdot g(Z)\le p(Z)$, otherwise go back to Step 1.

It can be shown that the output of the algorithm is distributed according to the density *p*. Moreover, the expected number of samples of (*Z*, *Y*) needed until a value of *Z* is accepted is equal to *c*. It is therefore desirable to choose *c* in (5.45) as small as possible. For a proof we refer to [29, Theorem 2.15].

### Transformation to a reference triangle

In this subsection we describe how to generate a sample of a random variable whose distribution depends on a specific triangle *T* of a given triangulation $\mathcal {T}_h$ by making use of a transformation to a reference triangle. The same approach is widely used in practice for the assembly of the stiffness matrix (1.4) and can therefore easily be added to existing code.

We purely focus on generating samples of the random variables $Y_{T,j}$, $T \in \mathcal {T}_h$, $j \in \{1,\ldots ,N_h\}$, introduced in Section 4. Recall that the probability density function associated to $Y_{T,j}$ is given by

$$\begin{aligned} p_{T,j}(x) = 3 |T|^{-1} \varphi _j(x) \mathbb {I}_{T}(x), \quad x \in {\mathcal {D}}\subset {\mathbb {R}}^2. \end{aligned}$$

Let us fix a triangle $T \in \mathcal {T}_h$ with vertices $(x_1,y_1)$, $(x_2,y_2)$ and $(x_3,y_3)$, such that $T \cap \textrm{supp}(\varphi _j)\ne \emptyset$. Without loss of generality we assume that $\varphi _j(x_1,y_1) = 1$.

We want to use the general rejection algorithm in order to generate samples of $Y_{T,j}$. However, the probability density function $p_{T,j}$ depends on the specific triangle and the basis function $\varphi _j$. Since it is inconvenient to set up the rejection method for each element and basis function separately, we will now describe in detail, how to simplify this problem by using a so called isoparametric transformation denoted by $\varGamma :T \rightarrow S_2$. Hereby, $S_2 \subset {\mathbb {R}}^2$ denotes the standard 2-simplex.

**Fig. 1:**
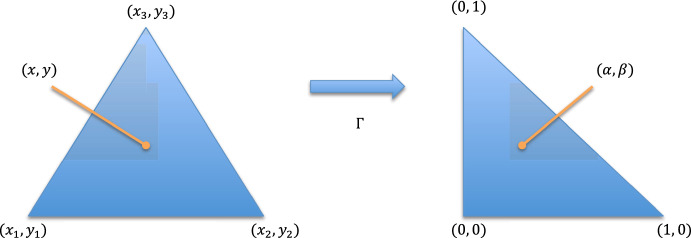
Triangle transformation to the standard 2-simplex, where (*x*, *y*) and $(\alpha ,\beta ) = \varGamma (x,y)$ represent interior points of the respective triangles

As illustrated in Figure 1 we denote the coordinates of a point in the given triangle *T* by (*x*, *y*), while the ones in the standard 2-simplex $S_2$ are written as $(\alpha ,\beta )$. Then, the coordinate transformation $\varGamma :T \rightarrow S_2$ is given by

$$\begin{aligned} \begin{bmatrix} \alpha \\ \beta \end{bmatrix} =\varGamma (x,y):= \begin{bmatrix} x_2-x_1 &  x_3-x_1\\ y_2-y_1 &  y_3-y_1\\ \end{bmatrix}^{-1} \begin{bmatrix} x-x_1\\ y-y_1 \end{bmatrix}, \end{aligned}$$

while the inverse $\varGamma ^{-1} :S_2 \rightarrow T$ is explicitly determined by

5.46 $$\begin{aligned} \begin{bmatrix} x\\ y \end{bmatrix} = \varGamma ^{-1}(\alpha ,\beta ) := \begin{bmatrix} x_2-x_1 &  x_3-x_1\\ y_2-y_1 &  y_3-y_1\\ \end{bmatrix} \begin{bmatrix} \alpha \\ \beta \end{bmatrix} + \begin{bmatrix} x_1\\ y_1 \end{bmatrix}. \end{aligned}$$

Observe that $$\varGamma ^{-1}(0,0) = (x_1,y_1)$$.

Next, we consider the mapping $\hat{\varphi } :S_2 \rightarrow {\mathbb {R}}$ defined by

5.47 $$\begin{aligned} \hat{\varphi }(\alpha ,\beta ) = 1 - \alpha - \beta , \quad \text {for all } (\alpha , \beta ) \in S_2. \end{aligned}$$

Since $\hat{\varphi }$ is affine linear one easily verifies that

$$\begin{aligned} \hat{\varphi }(\alpha ,\beta ) = \varphi _j( \varGamma ^{-1}(\alpha ,\beta )), \quad \text {for all } (\alpha , \beta ) \in S_2. \end{aligned}$$

Moreover, it holds

$$\begin{aligned} \int _{S_2} \hat{\varphi }(\alpha ,\beta ) \,\textrm{d}(\alpha ,\beta ) = \frac{1}{3}|S_2| = \frac{1}{6}. \end{aligned}$$

Therefore, the mapping $\hat{p} :{\mathbb {R}}^2 \rightarrow {\mathbb {R}}$ given by

5.48 $$\begin{aligned} \hat{p}(\alpha ,\beta ) = 6 \hat{\varphi }(\alpha ,\beta ) \mathbb {I}_{S_2}(\alpha ,\beta ), \quad \text {for } (\alpha , \beta ) \in {\mathbb {R}}^2, \end{aligned}$$

is a probability density function. Suppose that $\hat{Y} :\varOmega \rightarrow {\mathbb {R}}^2$ is a random variable with distribution $\hat{p}(\alpha ,\beta )\,\textrm{d}(\alpha ,\beta )$. Then, it follows that

$$\begin{aligned} Y_{T,j} \sim \varGamma ^{-1}( \hat{Y}), \end{aligned}$$

i.e. both random variables are identically distributed with the probability density function $p_{T,j}$. In fact, for every $B \in {\mathcal {B}}({\mathbb {R}}^2)$ it holds

$$\begin{aligned} {\mathbb {P}}( \{ \varGamma ^{-1}( \hat{Y}) \in B \} ) = {\mathbb {P}}( \{ \hat{Y} \in \varGamma (B) \} ) = \int _{\varGamma (B)} \hat{p}(\alpha ,\beta ) \,\textrm{d}(\alpha ,\beta ). \end{aligned}$$

After inserting $\hat{p}$ and since $\varGamma (B) \cap S_2 = \varGamma (B \cap T)$ we arrive at

$$\begin{aligned} {\mathbb {P}}( \{ \varGamma ^{-1}( \hat{Y}) \in B \} )&= 6 \int _{\varGamma (B)} \hat{\varphi }(\alpha ,\beta ) \mathbb {I}_{S_2}(\alpha ,\beta ) \,\textrm{d}(\alpha ,\beta ) = 6 \int _{\varGamma (B \cap T)} \hat{\varphi }(\alpha ,\beta ) \,\textrm{d}(\alpha ,\beta )\\&= 6 \int _{B \cap T} \hat{\varphi }( \varGamma (x,y) ) |\det (D\varGamma )(x,y)| \,\textrm{d}(x,y)\\&= 6 \int _{B} \varphi _j( x,y) \mathbb {I}_T(x,y) |\det (D\varGamma )(x,y)| \,\textrm{d}(x,y) \end{aligned}$$

by a change of coordinates. Since $\varGamma$ is affine linear, the Jacobian $D \varGamma \in {\mathbb {R}}^{2,2}$ is constant and the determinant is easily computed as

$$\begin{aligned} |\det (D\varGamma )| = |\det (D\varGamma ^{-1})|^{-1} = \frac{1}{2|T|}. \end{aligned}$$

Therefore,

$$\begin{aligned} {\mathbb {P}}( \{ \varGamma ^{-1}( \hat{Y}) \in B \} ) = \frac{3}{|T|} \int _{B} \varphi _j( x,y) \mathbb {I}_T(x,y) \,\textrm{d}(x,y) = \int _B p_{T,j}(x,y) \,\textrm{d}(x,y). \end{aligned}$$

Consequently, in order to generate a sample of the random variable $Y_{T,j} \sim p_{T,j}$ it is sufficient to generate a sample of $\hat{Y} \sim \hat{p}$ and to apply the transformation $\varGamma ^{-1}$.

In addition, for the cases of $\varphi _j(x_2,y_2)=1$ or $\varphi _j(x_3,y_3)=1$, if using the same triangle transform as illustrated in Figure 1, the only step that differs from the above description is in (5.47). It needs to be changed accordingly to

$$\begin{aligned} \hat{\varphi }(\alpha ,\beta ) = \alpha , \quad \text {for all } (\alpha , \beta ) \in S_2, \end{aligned}$$

if $$\varphi _j(x_2,y_2)=1$$, or

$$\begin{aligned} \hat{\varphi }(\alpha ,\beta ) = \beta , \quad \text {for all } (\alpha , \beta ) \in S_2, \end{aligned}$$

in the case of $\varphi _j(x_3,y_3)=1$.

### Generating samples of $\hat{Y}$ on the reference triangle

Next, we discuss how to generate samples of the random variable $\hat{Y}$ introduced in (5.48). To this end, we recall that $\hat{Y} \sim \hat{p}(\alpha ,\beta )\,\textrm{d}(\alpha ,\beta )$. We apply the general rejection algorithm from Section 5.1 with

$$\begin{aligned} g(\alpha ,\beta )=2\mathbb {I}_{S_2}(\alpha ,\beta ), \quad \text {for } (\alpha ,\beta ) \in {\mathbb {R}}^2, \end{aligned}$$

as the probability density function of the random variable *Z*, i.e. $Z \sim \mathcal {U}(S_2)$. As above $S_2 \subset {\mathbb {R}}^2$ denotes the standard 2-simplex. We also define

$$\begin{aligned} c:= \sup \Big \{\frac{\hat{p}(\alpha ,\beta )}{g(\alpha ,\beta )}\, | \, (\alpha ,\beta ) \in S_2\Big \} = \sup \Big \{ \frac{1}{2} \hat{p}(\alpha ,\beta ) \, | \, (\alpha ,\beta ) \in S_2\Big \} = 3. \end{aligned}$$

Then, (5.45) is satisfied. Therefore, the general rejection algorithm is applicable and generates samples of $\hat{Y} \sim \hat{p}(\alpha ,\beta )\,\textrm{d}(\alpha ,\beta )$ as follows:

1. Generate $Z = (Z_1,Z_2) \sim \mathcal {U}(S_2)$ as follows:
  - Generate $$U_1, U_2 \sim \mathcal {U}(0,1)$$ independently.
  - If $U_1 + U_2 \le 1$ then set $Z:= (U_1,U_2)$, else set $Z:= (1-U_1, 1 -U_2)$.
2. Generate $Y \sim \mathcal {U}(0,c)$ independently of *Z*.
3. Output $Z = (Z_1,Z_2)$ if $Y g(Z_1,Z_2) \le \hat{p}(Z_1,Z_2)$, else go back to Step 1.

#### Remark 3

As an alternative to the rejection method one could generate samples of $\hat{Y}= (\hat{Y}_1, \hat{Y}_2)$ by first applying the inversion method, cf. [29, Chapter 2], for the simulation of the marginal distribution of the first variable $\hat{Y}_1$. Thereafter, a further application of the inversion method can be used to generate a sample of $\hat{Y}_2$ conditional on the already generated sample of $\hat{Y}_1$. Depending on the actual implementation, this could be more efficient. However, this approach is much harder to generalize to other probability density functions or to higher dimensional domains.

### Generating uniformly distributed samples on arbitrary elements

In this subsection, we briefly discuss the generation of uniformly distributed random variables $Z_T \sim \mathcal {U}(T)$ for an arbitrary triangle $T \in \mathcal {T}_h$. These random variables are required for the randomized quadrature formula (3.19). This is easily accomplished by making use of the results from the previous two subsections. Indeed, we just have to generate a sample of a uniformly distributed random variable $Z \sim \mathcal {U}(S_2)$, where $S_2$ again denotes the 2-simplex. Then, we apply the corresponding inverse transformation $\varGamma ^{-1}$ from (5.46) associated to the given triangle $T \in \mathcal {T}_h$. As a result, we obtain $Z_T = \varGamma ^{-1}(Z) \sim \mathcal {U}(T)$ for $T \in \mathcal {T}_h$.

The sampling procedure is summarized in the following two steps.

1. Generate $Z = (Z_1,Z_2) \sim \mathcal {U}(S_2)$ as follows:
  - Generate $$U_1, U_2 \sim \mathcal {U}(0,1)$$ independently.
  - If $U_1 + U_2 \le 1$ then set $Z:= (U_1,U_2)$, else set $Z:= (1-U_1, 1 -U_2)$.
2. Output: $Z_T = \varGamma ^{-1}(Z_1,Z_2)$, where $\varGamma ^{-1}$ in (5.46) uses the coordinates of the vertices of *T*.

### Implementation of the FEM with randomized quadrature formulas

In this part, we illustrate the implementation of the finite element method with the randomized quadrature formula (3.19) for the elliptic equation (1.1). The implementation of (4.42), which is based on the importance sampling estimator, can be done in a similar way.

Algorithm 1 lists one possibility to compute a realization of the numerical approximation of the solution to (1.1) based on the Monte Carlo estimator (3.19).

**Algorithm 1:**
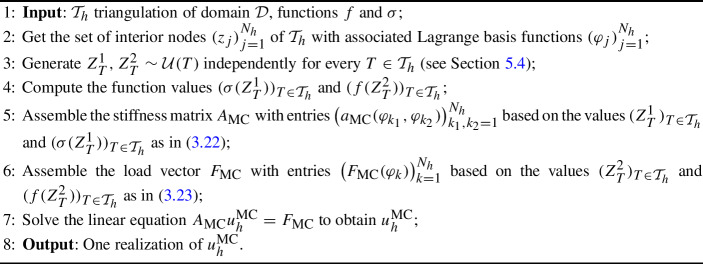
FEM with MC estimator (3.19) for the elliptic equation (1.1)

Observe in Step 5 that one only has to sum over those triangles in (3.22), which are contained in the joint support of the basis functions $\varphi _{k_1},\varphi _{k_2}$. Hence, the sum in (3.22) consists of at most two non-zero terms if $k_1\ne k_2$. In particular, the stiffness matrix $A_{\text {MC}}$ remains sparse and the complexity of assembling $A_{\text {MC}}$ grows only linearly with $N_h$. In addition, the matrix $A_h$ remains positive definite and allows the application of linear solvers for large sparse systems as described in, e.g., [17].

## Numerical experiments

This section is devoted to numerical experiments, illustrating the performance of the randomized quadrature formulas based on the MC estimator (3.19) and/or the IS estimator (4.37).

To this end, on the domain $\mathcal {D}= (0, 1)^2 \subset {\mathbb {R}}^2$, we consider in the first experiment example the Poisson equation (4.41) with homogeneous Dirichlet boundary conditions, focusing on the performance of both estimators in approximating the load vectors; and in the second one the general elliptic equations with homogeneous Dirichlet boundary conditions, focusing on the performance of the MC estimator in approximating the stiffness matrix. In each example, we also briefly compare the performance of the randomized quadrature formula with the deterministic *barycentric quadrature rule* (BQR), which is also known as a one-point Gaussian quadrature formula. We refer to [22] and [28, Section 5.6].

### The general set-up

For the finite element method we choose a family of structured uniform meshes. To be more precise, the domain ${\mathcal {D}}$ is first subdivided into squares with uniform mesh size $h = 2^{-n}$, $n\in \{2,\ldots ,8\}$. Then, we obtain the triangulation $\mathcal {T}_h$ by bisecting each square along the diagonal from the upper left to the lower right vertex. As in the previous sections, the shape functions are chosen to be piecewise linear. For each fixed triangulation $\mathcal {T}_h$ we then solve the discrete problems (3.24) and/or (4.42) as sketched in Algorithm 1. As above, we denote the corresponding discrete solutions by $u^{\text {MC}}_h$ and $u^{IS}_h$, respectively.

To justify the performance of the two randomized quadrature formulas, we focus on the distances (in $L^2(\varOmega ;H^1({\mathcal {D}}))$-semi-norm and $L^2(\varOmega ;L^2({\mathcal {D}}))$-norm) between the discrete solutions $u^{\text {MC}}_h$ and/or $u^{IS}_h$ and the standard finite element solution $u_h = R_h u$. Recall that the computation of the $H^1({\mathcal {D}})$-semi-norm is easily accomplished in practice by making use of the relationship

6.49 $$\begin{aligned} |v_h|^2_{H^1({\mathcal {D}})} = a(v_h,v_h) = \sum _{i,j = 1}^{N_h} v_i v_j a( \varphi _i, \varphi _j) = \textbf{v}^\top A_h \textbf{v}, \end{aligned}$$

for every $v_h = \sum _{j = 1}^{N_h} v_j \varphi _j \in S_h$ with $\textbf{v} = [v_1,\ldots ,v_{N_h}]^{\top } \in {\mathbb {R}}^{N_h}$. Similarly, $|v_h|^2_{L^2({\mathcal {D}})}$ is obtained if the stiffness matrix $A_h$ is replaced by the mass matrix $M_h = [(\varphi _i,\varphi _j)_{L^2({\mathcal {D}})}]_{i,j=1}^{N_h}$ in (6.49). Based on those observations, one can get Monte Carlo approximations of the errors $\Vert u^{\text {MC}}_h-u_h\Vert _{L^2(\varOmega ;H^1_0({\mathcal {D}}))}$ and $\Vert u^{IS}_h-u_h\Vert _{L^2(\varOmega ;H^1_0({\mathcal {D}}))}$, achieved by generating $M = 10^4$ independent realizations of the random variables $u^{\text {MC}}_h$ and $u^{IS}_h$ and taking suitable averages.

### Example: the Poisson equation with different forcing terms

In this example, we consider the Poisson equation which satisfies (1.3) with $\sigma \equiv 1$ and is subject to different forcing terms $f_1$ or $f_2$. The first forcing term $f_1$ is singular but still square-integrable, defined by

6.50 $$\begin{aligned} f_1(x,y):=|x-y|^{-q} + 10\sin (2^3 \pi x) \text {sgn}(2y-x), \quad \text {for } (x,y) \in {\mathcal {D}}, \end{aligned}$$

with $q=0.49$ and $\textrm{sgn} :{\mathbb {R}}\rightarrow {\mathbb {R}}$ given by

$$\begin{aligned} \text{ sgn }(x):= \begin{aligned} {\left\{ \begin{array}{ll} -1,&  \ \text{ if } x<0,\\ 0,&  \ \text{ if } x=0,\\ 1,&  \ \text{ if } x>0. \end{array}\right. } \end{aligned} \end{aligned}$$

The second forcing term $f_2 :{\mathcal {D}}\rightarrow {\mathbb {R}}$ is taken more regular by setting

6.51 $$\begin{aligned} f_2(x,y) := 8 x(1-x)y(1-y), \quad \text {for } (x,y) \in {\mathcal {D}}. \end{aligned}$$

In fact, it can be easily verified that $f_2 \in H^1_0({\mathcal {D}}) \cap H^2({\mathcal {D}})$.

Considering Poisson equation allows us to neglect the approximation error $u_h - u$ stemming from the finite element method itself. More precisely, we first take note of the fact that ${\mathbb {E}}[u_h^{\text {MC}}] = {\mathbb {E}}[u_h^{IS}] = u_h$. In fact, since $\sigma \equiv 1$ we have that $a_{\text {MC}} = a$ in (3.24). Hence, after taking expectation in (3.24) and since $Q_{\text {MC}}$ is unbiased we obtain that

$$\begin{aligned} a\big ( {\mathbb {E}}[ u_h^{\text {MC}} ], v_h \big ) = {\mathbb {E}}\big [ a_{\text {MC}}(u_h^{\text {MC}}, v_h) \big ] = {\mathbb {E}}\big [ F_{\text {MC}}(v_h) \big ] = F(v_h) \end{aligned}$$

for every $v_h \in S_h$. Therefore, the function ${\mathbb {E}}[u_h^{\text {MC}}] \in S_h$ is a solution to (1.3), i.e. ${\mathbb {E}}[u_h^{\text {MC}}] = u_h$ for every $h \in (0,1]$. The same arguments apply to ${\mathbb {E}}[u_h^{IS}]$. This motivates to replace $u_h$ in the error computation by the Monte Carlo means

$$\begin{aligned} u_h \approx \frac{1}{M} \sum _{i = 1}^M u_{h,i}^{\text {MC}}, \quad \text { and } u_h \approx \frac{1}{M} \sum _{i = 1}^M u_{h,i}^{IS}, \end{aligned}$$

where $(u_{h,i}^{\text {MC}})_{i = 1}^M$ and $(u_{h,i}^{IS})_{i = 1}^M$ denote families of independent and identically distributed copies of $u_h^{\text {MC}}$ and $u_h^{IS}$, respectively.

**Fig. 2:**
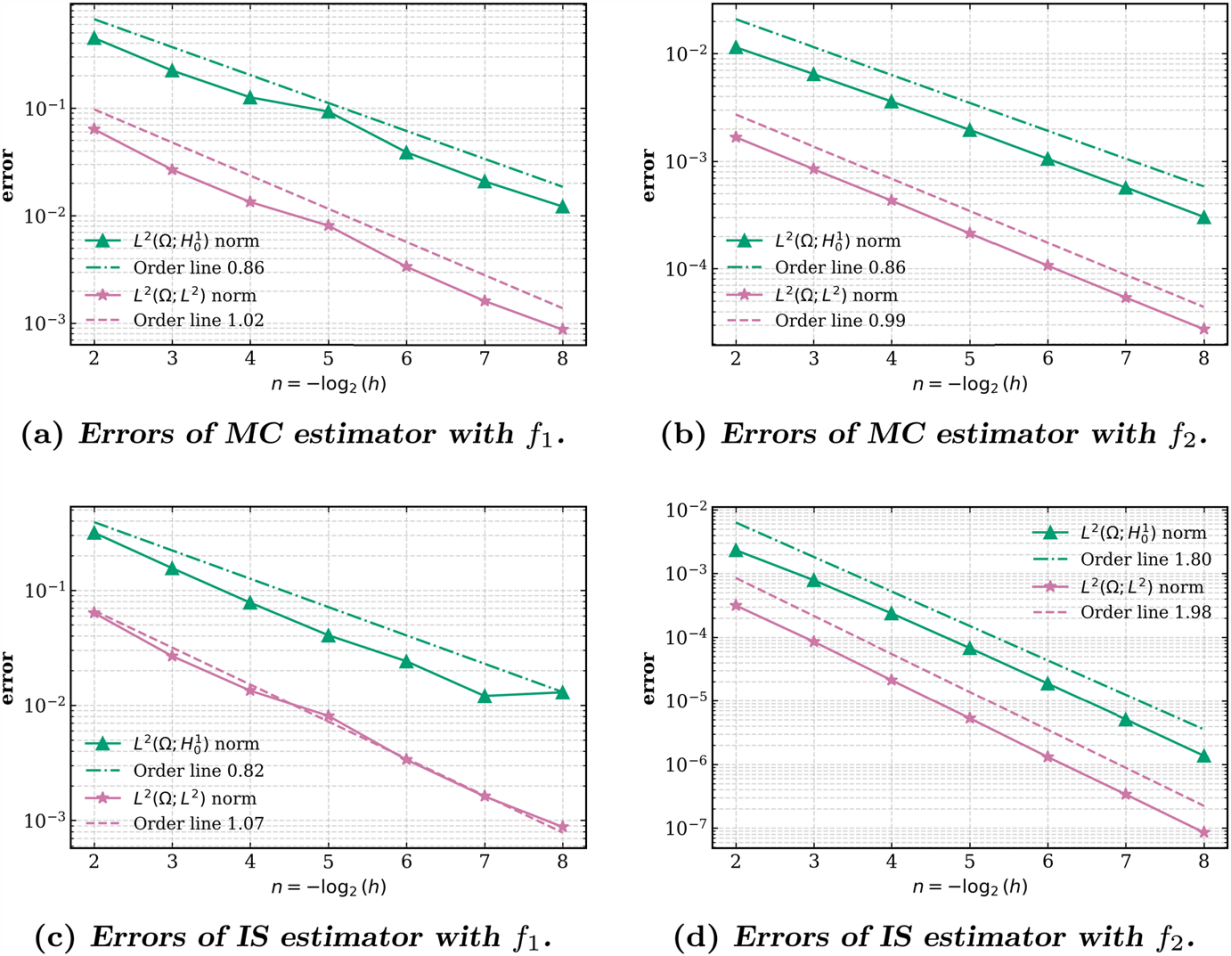
Error plots of the MC estimator (3.19) and IS estimator (4.37) for the Poisson equation (4.41) with singular forcing term $f_1$ and smooth forcing term $f_2$

Figure 2 shows the results of experiments using MC estimator and IS estimator. In each of the four subfigures the Monte Carlo approximations of the $L^2(\varOmega ;H^1_0({\mathcal {D}}))$-norm and the $L^2(\varOmega ;L^2({\mathcal {D}}))$-norm of the errors $u_h^{\text {MC}} - u_h$ and $u_h^{IS} - u_h$ are plotted versus the mesh size $h = 2^{-n}$, $n \in \{2,\ldots ,8\}$. Hereby, the first two subfigures show the corresponding errors for the MC estimator (3.19) applied to the Poisson equation with the forcing terms $f_1$ and $f_2$ defined in (6.50) and (6.51), respectively. As it can be seen from the order lines, the errors decay approximately with orders roughly 0.86 and 1. Given that $f_1$ is singular and only square-integrable, the experimental order of convergence is therefore larger than it is predicted by Theorem 2.

In Figures 2 **(c)** and **(d)** we see the corresponding results for the IS estimator (4.37). While the values in Figure 2 **(c)** are comparable to those in Figure 2 **(a)**, it can be seen from Figure 2 **(d)** that the IS estimator benefits considerably from the additional smoothness of $f_2$. In fact, the experimental order of convergence is close to 2 in Figure 2 **(d)**, which is in line with the results in Theorem 5.

**Fig. 3:**
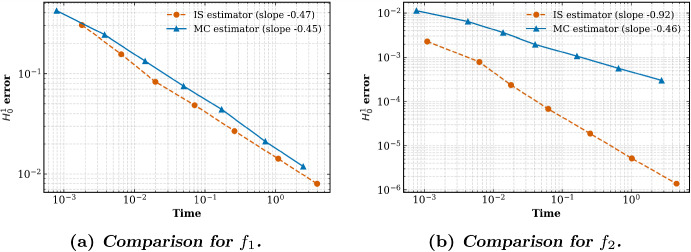
Computational time versus errors in $L^2(\varOmega ;H^1_0({\mathcal {D}}))$-norm of the MC estimator (3.19) and IS estimator (4.37) with singular forcing term $f_1$ and smooth forcing term $f_2$

In Figure 3, we plot the estimated values of the errors in the $L^2(\varOmega ;H^1_0({\mathcal {D}}))$-norm versus the computational time. This allows a better comparison of the performance of the two randomized quadrature rules since the IS estimator is computational more expensive due to the application of the general rejection method. Hereby, the computational time is taken as the average time needed to assemble the load vector $f_h \in {\mathbb {R}}^{N_h}$ for $f_1$ or $f_2$ with either (3.19) or (4.37). More precisely, we only measured the time of Step 6 in Algorithm 1. The other steps are neglected, since they are essentially independent of the choice of the randomized quadrature formula.

As it can be seen in both subfigures, the importance sampling estimator (4.37) is superior to the MC estimator. For both forcing terms the higher computational cost is offset by the better accuracy of the IS estimator (4.37). In particular, this is true for the smooth forcing term $f_2$ due to the better experimental order of convergence of (4.37). On the other hand, it is not very pronounced for the singular forcing term $f_1$ as can be seen in Figure 3 **(a)**.

Finally, let us also briefly compare the performance of the randomized quadrature formula with BQR. Table 1 lists the corresponding estimates of the errors stemming from the application of the deterministic quadrature rule. Hereby, the errors are measured with respect to the semi-norm in $H^1({\mathcal {D}})$. Apparently, BQR is not useful for approximating the load vector involving the singular forcing term $f_1$.

This is easily explained by the geometry of the triangulation $\mathcal {T}_h$. For every mesh size $h = 2^{-n}$ there always exist triangles in $\mathcal {T}_h$ whose barycenters lie on the diagonal in ${\mathcal {D}}$, where $f_1$ is singular. To avoid NaN entries in the load vector we replaced $f_1$ by the modification

$$\begin{aligned} \tilde{f}_1(x,y) := (\textrm{eps} + |x-y|)^{-q} + 10\sin (2^3 \pi x) \text {sgn}(2y-x), \quad \text {for } (x,y) \in {\mathcal {D}}, \end{aligned}$$

where $\textrm{eps}$ is equal to the machine precision (in Matlab^©^$\textrm{eps} \approx 2.2204 \times 10^{-16}$). Nevertheless, the discretization errors indicate that BQR is not reliable for applications with singular forcing terms. This can only be circumvented by adapting the mesh to avoid point evaluations close to singularities of the given forcing term. However, this requires a priori knowledge of the position of the singularities or adaptive methods for their automatic detection when generating the mesh. The randomized quadrature formulas, on the other hand, lead to a robustification of the finite element method based on rudimentary uniform meshes without using any preknowledge of the forcing term.

**Table 1:** Discretization errors of the (deterministic) barycentric quadrature rule applied to (4.41) with $f_1$

mesh size $h^{-n}$	$n=3$	$n=4$	$n=5$	$n=6$	$n=7$	$n=8$
error in $H^1_0({\mathcal {D}})$-norm	1.4e+6	7.7e+5	4.0e+5	2.1e+5	1.0e+5	5.2e+4

### Example: elliptic equations with different $\sigma$

**Fig. 4:**
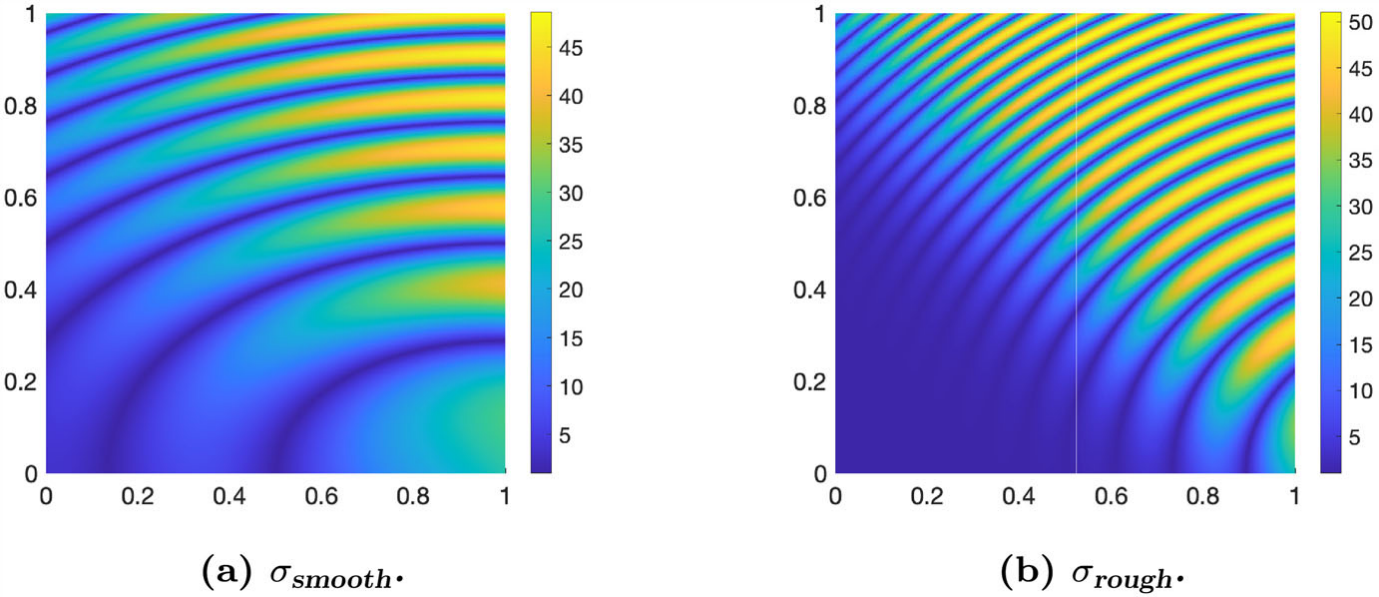
The profiles of two $\sigma (x,y)$ functions used in the simulation of the elliptic equation at resolution $n=8$

In this example, we introduce two $\sigma$ functions with different regularity, both of which are in the form of

$$\begin{aligned} \sigma (x,y;\theta ,c):=\sigma _1(x,y; \theta _1)\sigma _2(x,y; \theta _2,\theta _3,\theta _4)+c, \end{aligned}$$

and consider a smooth forcing term $f_2$ defined in (6.51). Here the sigmoid function $0 \le \sigma _1(x,y;\theta _1) \le 50$ is to introduce a smooth or sharp gradient at the interface $x+y-1$, defined as

$$ \sigma _1(x,y;\theta _1) = \frac{50}{ 1 + \textrm{e}^{-\theta _1(x+y-1)}}, $$

for a parameter $\theta _1>0$ that controls the magnitude of the gradient of $\sigma _1$ at the interface, and a decaying ripple feature is captured in

$$ \sigma _2(x,y;\theta _2,\theta _3,\theta _4) = \Bigl | \cos \bigl [ 2\pi (\theta _2 x - \theta _3 x^2 - \theta _4 y^2 \bigr ) \bigr ] \Bigr | , $$

for some positive parameters $\theta _2,\theta _3,\theta _4$ controlling the initial frequency of the ripple, its decaying rate, and rotation respectively. Let $c=1$ be an offset to impose positivity in the function, then a smooth sigma can be obtained by

$$\begin{aligned} \sigma _{\text {smooth}}(x,y) := \sigma (x,y; \theta =(3,2,1,3)) \end{aligned}$$

while another realisation with a more rough profile - higher gradient magnitudes profile can be obtained by

$$\begin{aligned} \sigma _{\text {rough}}(x,y) := \sigma (x,y; \theta =(9,8,3,5)) \end{aligned}$$

as depicted in figure 4.

For each combination of $\sigma$ (from $\sigma _{\text {rough}}$ and $\sigma _{\text {smooth}}$) and $f=f_2$ we apply the Monte Carlo estimator (3.19) and solve the finite element problem defined in (3.24), using the classical FEM with the BQR as a benchmark. As the gradient of basis $\varphi _i$ being constant on triangle elements, the difference in approximating the stiffness matrix in (1.5) is highlighted by the estimator’s ability to approximate integrals of $\sigma$ functions. That is,

$$\begin{aligned} \int _{\mathcal {D}}\sigma (x) \nabla \varphi _i(x) \cdot \nabla \varphi _j (x) \,\textrm{d}x&=\sum _{T\in \mathcal {T}}\int _T \sigma (x) \nabla \varphi _i(x) \cdot \nabla \varphi _j (x) \,\textrm{d}x\\&= \sum _{T\in \mathcal {T}} (\nabla \varphi _i(C_T) \cdot \nabla \varphi _j (C_T))\int _T \sigma (x) \,\textrm{d}x, \end{aligned}$$

where $C_T$ is the centroid of *T*. To approximate $\int _T \sigma (x) \,\textrm{d}x$, both estimators use single value evaluation $|T|\sigma (x_T)$: the MC estimator (3.19) uses a random sample on *T* as $x_T$ while the BQR uses $x_T=C_T$.

**Fig. 5:**
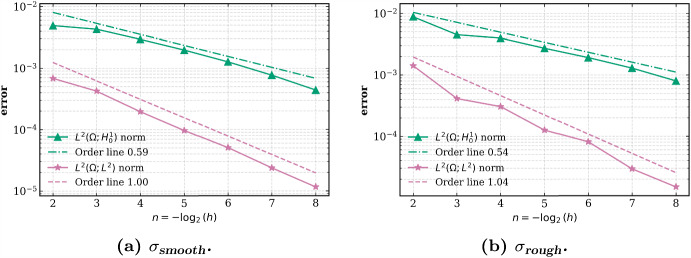
Error plots of the MC estimator (3.19) for the Elliptic equation (1.1) with $f_2$ and different choices for $\sigma$

Figure 5 demonstrate the results of experiments using MC estimator (3.19) with varying regularity of $\sigma$. In each of subfigures the Monte Carlo approximations of the $L^2(\varOmega ;H^1_0({\mathcal {D}}))$-norm and the $L^2(\varOmega ;L^2({\mathcal {D}}))$-norm of the errors $u_h^{\text {MC}} - u_h$ are plotted versus the mesh size $h = 2^{-n}$, $n \in \{2,\ldots ,8\}$. The convergence plots illustrate that the numerical scheme achieves robust convergence rates for both coefficients, exhibiting an order-one convergence in the $L^2(\varOmega ;L^2({\mathcal {D}}))$ norm and an order-half convergence in the $L^2(\varOmega ;H^1_0({\mathcal {D}}))$ norm, where the first one is in general an open question for the future research. While the convergence order is preserved, the error magnitude clearly indicates that the rough coefficient, $\sigma _{\text {rough}}$, results in a significantly higher magnitude of the error across both norms compared to the smooth coefficient, $\sigma _{\text {smooth}}$, for any fixed mesh resolution. For $\sigma _{\text {rough}}$, a crucial observation is the imperfect alignment of the measured error points with the theoretical order line, indicating a greater influence of pre-asymptotic behavior and higher error constants *C* compared to the $\sigma _{\text {smooth}}$ case.

**Fig. 6:**
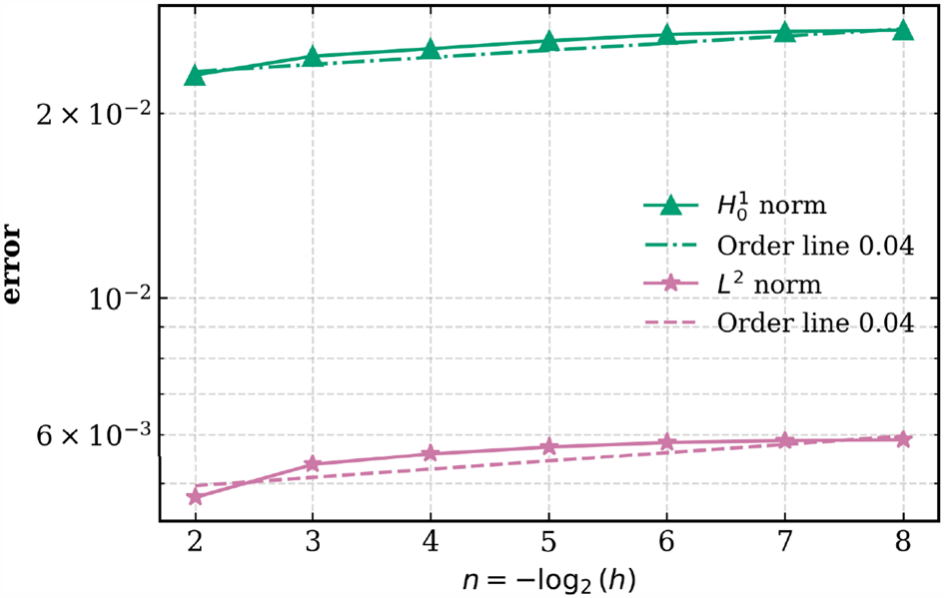
Error plot of the BQR for the Elliptic equation (1.1) with $f_2$ and $\sigma _{\text {smooth}}$

For both cases, BQR shows divergence. Figure 6 demonstrates that employing BQR severely degrades the performance of the numerical scheme, even with smooth coefficients. This degradation occurs because the low precision of BQR introduces a quadrature error that dominates the total discretization error, preventing the method from achieving its full theoretical convergence orders in both the solution and the gradient norms.

## An alternative estimate for Lemma 3

### Lemma 6

Let Assumption 2 be satisfied. Then there exists $C \in (0,\infty )$ such that for every $h \in (0,1]$, $f \in L^2({\mathcal {D}})$ and $S_h$-valued random variable $v_h \in L^2(\varOmega ;H^1_0({\mathcal {D}}))$ it holds

$$\begin{aligned} \big | {\mathbb {E}}\big [ F_{\text {MC}}(v_h) - F(v_h) \big ] \big | \le \Vert f\Vert _{L^2({\mathcal {D}})} \big (2 h \Vert v_h \Vert _{L^2(\varOmega ;H^1_0({\mathcal {D}}))} + \Vert v_h - {\mathbb {E}}[v_h] \Vert _{L^2(\varOmega ;L^2({\mathcal {D}}))}\big ). \end{aligned}$$

### Proof

First, we collect some observations: Due to Lemma 1 we have

$$\begin{aligned} {\mathbb {E}}\big [ F(v_h) \big ] = F\big ({\mathbb {E}}[v_h]\big ) = {\mathbb {E}}\big [ F_{MC}({\mathbb {E}}[v_h]) \big ]. \end{aligned}$$

Hence, we have

$$\begin{aligned} \big | {\mathbb {E}}\big [ F_{\text {MC}}(v_h) - F(v_h) \big ] \big |&= \big | {\mathbb {E}}\big [ F_{\text {MC}}(v_h) - F_{\text {MC}}({\mathbb {E}}[v_h]) \big ] \big |\\&= \Big | \sum _{T \in \mathcal {T}_h} |T| {\mathbb {E}}\big [ f(Z_T) \big (v_h(Z_T) - {\mathbb {E}}[v_h(Z_T)] \big ) \big ] \Big |\\&\le \Big ( \sum _{T \in \mathcal {T}_h} |T| {\mathbb {E}}\big [ |f(Z_T)|^2 \big ] \Big )^{\frac{1}{2}}\\&\quad \times \Big ( \sum _{T \in \mathcal {T}_h} |T| {\mathbb {E}}\big [ \big |v_h(Z_T) - {\mathbb {E}}[v_h(Z_T)] \big |^2 \big ] \Big )^{\frac{1}{2}}, \end{aligned}$$

where we also applied the Cauchy–Schwarz inequality. Next, let us observe that

$$\begin{aligned} \Big ( \sum _{T \in \mathcal {T}_h} |T| {\mathbb {E}}\big [ |f(Z_T)|^2 \big ] \Big )^{\frac{1}{2}} = \Vert f \Vert _{L^2({\mathcal {D}})}. \end{aligned}$$

By $C_T \in T$ we denote the barycenter of the triangle *T*. Then, an application of the triangle inequality yields

$$\begin{aligned}&\Big ( \sum _{T \in \mathcal {T}_h} |T| {\mathbb {E}}\big [ \big |v_h(Z_T) - {\mathbb {E}}[v_h(Z_T)] \big |^2 \big ] \Big )^{\frac{1}{2}}\\&\quad \le \Big ( \sum _{T \in \mathcal {T}_h} |T| {\mathbb {E}}\big [ \big |v_h(Z_T) - v_h(C_T) \big |^2 \big ] \Big )^{\frac{1}{2}} \\&\qquad + \Big ( \sum _{T \in \mathcal {T}_h} |T| {\mathbb {E}}\big [ \big |v_h(C_T) - {\mathbb {E}}[v_h(C_T)] \big |^2 \big ] \Big )^{\frac{1}{2}} \\&\qquad + \Big ( \sum _{T \in \mathcal {T}_h} |T| \big |{\mathbb {E}}[v_h(C_T) - v_h(Z_T)] \big |^2 \Big )^{\frac{1}{2}} \\&\quad \le 2 \Big ( \sum _{T \in \mathcal {T}_h} |T| {\mathbb {E}}\big [ \big |v_h(Z_T) - v_h(C_T) \big |^2 \big ] \Big )^{\frac{1}{2}} \\&\qquad + \Big ( \sum _{T \in \mathcal {T}_h} |T| {\mathbb {E}}\big [ \big |v_h(C_T) - {\mathbb {E}}[v_h(C_T)] \big |^2 \big ] \Big )^{\frac{1}{2}} \\&\quad =: \varGamma _1 + \varGamma _2. \end{aligned}$$

For $\varGamma _1$ we recall that $v_h \in L^2(\varOmega ;S_h)$. Thus, $x \mapsto \nabla v_h(x)$ is constant on each triangle almost surely. In addition, we have $|Z_T - C_T| \le h$. Together, this yields

$$\begin{aligned} \varGamma _1&= 2 \Big ( \sum _{T \in \mathcal {T}_h} |T| {\mathbb {E}}\big [ \big |v_h(Z_T) - v_h(C_T) \big |^2 \big ] \Big )^{\frac{1}{2}} \\&= 2 \Big ( \sum _{T \in \mathcal {T}_h} |T| {\mathbb {E}}\big [ \big | \nabla v_h(C_T) \cdot (Z_T - C_T) \big |^2 \big ] \Big )^{\frac{1}{2}} \\&\le 2 h \Vert v_h \Vert _{L^2(\varOmega ; H^1_0({\mathcal {D}}))}. \end{aligned}$$

For $\varGamma _2$ we make use of the fact that

1.52 $$\begin{aligned} \int _T w_h(x) \,\textrm{d}x = |T| w_h(C_T) \end{aligned}$$

which holds for every affine-linear mapping $w_h$ on *T*. From this it follows that

$$\begin{aligned} v_h(C_T) - {\mathbb {E}}[v_h(C_T)] = |T|^{-1} \int _T (v_h(x) - {\mathbb {E}}[v_h(x)]) \,\textrm{d}x. \end{aligned}$$

Therefore, it holds

$$\begin{aligned} \varGamma _2&= \Big ( \sum _{T \in \mathcal {T}_h} |T| {\mathbb {E}}\big [ \big |v_h(C_T) - {\mathbb {E}}[v_h(C_T)] \big |^2 \big ] \Big )^{\frac{1}{2}} \\&= \Big ( \sum _{T \in \mathcal {T}_h} |T|^{-1} {\mathbb {E}}\Big [ \Big | \int _T \big (v_h(x) - {\mathbb {E}}[v_h(x)]\big ) \,\textrm{d}x \Big |^2 \Big ] \Big )^{\frac{1}{2}} \\&\le \Big ( \sum _{T \in \mathcal {T}_h} {\mathbb {E}}\Big [ \int _T \big |v_h(x) - {\mathbb {E}}[v_h(x)]\big |^2 \,\textrm{d}x \Big ] \Big )^{\frac{1}{2}} \\&= \big \Vert v_h - {\mathbb {E}}[v_h]\big \Vert _{L^2(\varOmega ;L^2({\mathcal {D}}))}. \end{aligned}$$

$$\square $$

### Remark 4

The new version of Lemma 6 is only useful, if we can prove an estimate for

$$\begin{aligned} \Vert u_h^\text {MC} - {\mathbb {E}}[u_h^{MC}] \Vert _{L^2(\varOmega ;L^2({\mathcal {D}}))}. \end{aligned}$$

In Theorem 2 we apply Lemma 3 with $v_h = \theta = u_h^{\text {MC}} - R_h u$.
